# Supplementary material for: FK506 bypasses the effect of erythroferrone in cancer cachexia skeletal muscle atrophy
Source: Cell Rep Med. 2023 Dec 4;4(12):101306. doi: 10.1016/j.xcrm.2023.101306 (PMC10772350; doi:10.1016/j.xcrm.2023.101306)
Supplement: Document S1. Figures S1–S6 and Table S1 [file mmc1.pdf]

Cell Reports Medicine, Volume *4*

Supplemental information

FK506 bypasses the effect of erythroferrone in cancer cachexia skeletal muscle atrophy

Erica Mina, Elisabeth Wyart, Roberta Sartori, Elia Angelino, Ivan Zaggia, Valentina Rausch, Mara Maldotti, Alessia Pagani, Myriam Y. Hsu, Alberto Friziero, Cosimo Sperti, Alessio Menga, Andrea Graziani, Emilio Hirsch, Salvatore Oliviero, Marco Sandri, Laura Conti, Léon Kautz, Laura Silvestri, and Paolo E. Porporato

Supplementary Figure 1

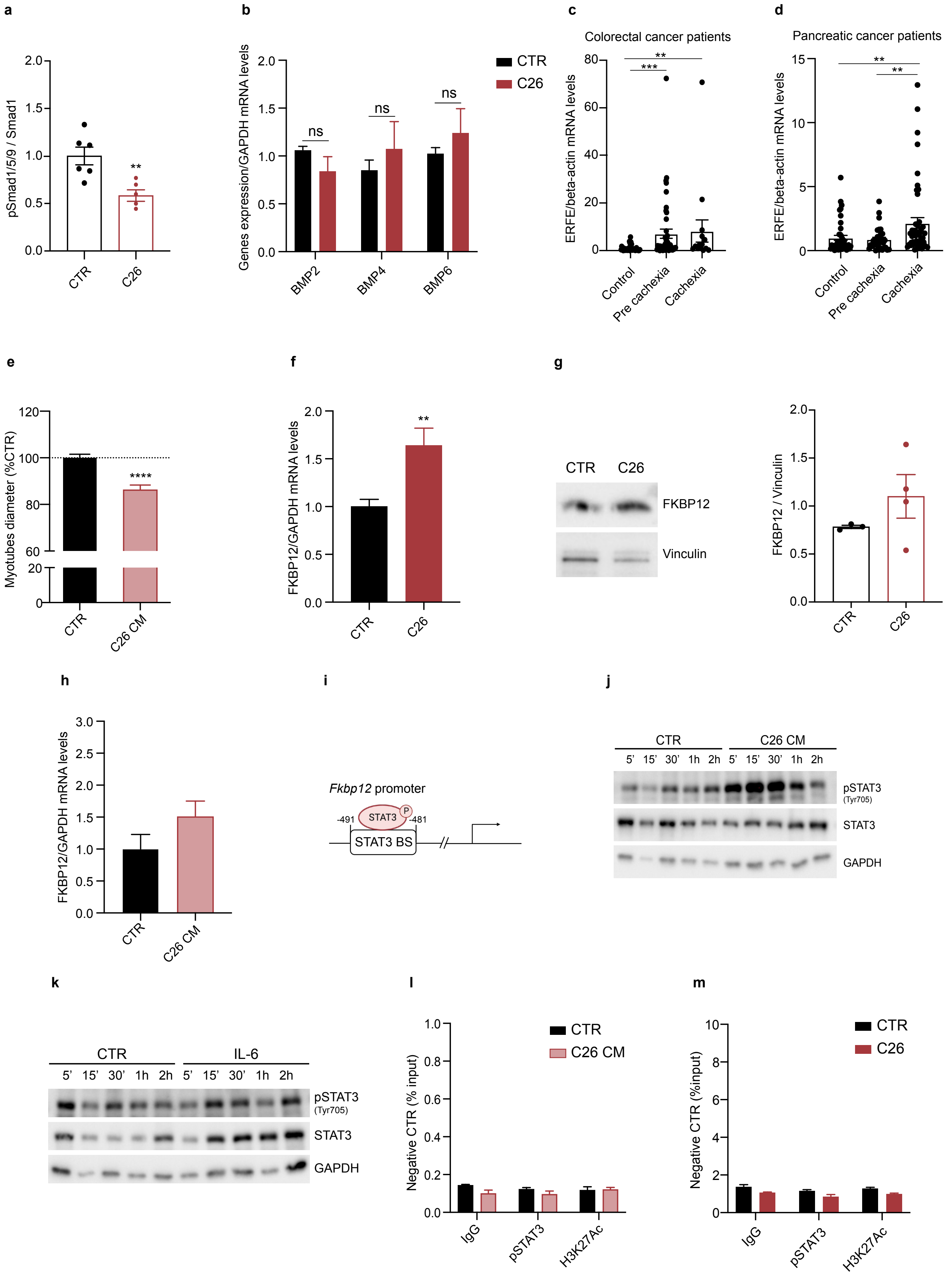

Supplemental information

Figure S1: The BMP-scavenger erythroferrone (ERFE) is upregulated in cachexia through STAT3 activation. Related to Fig. 1.

a) Densiometric quantification of the immunoblot of pSmad1/5/8 and Smad1 in the quadriceps of control and C26 tumor-bearing mice. (n=5/6) **b)** Expression levels of BMP2 (n=4/5), BMP4 (n=4) and BMP6 (n=9), in the gastrocnemii of control and C26 tumor-bearing mice. **c)** Expression levels of *ERFE* relative to beta actin in human skeletal muscle biopsies from pre-cachectic and cachectic colorectal cancer patients compared to control individuals (Ctr, n=41; pre-cachectic, n=45; cachectic, n=15; control vs pre-cachexia q value=0.0001; control vs cachexia q value=0.0016). Values are reported as fold change over control group. **d)** Expression levels of *ERFE* relative to beta actin in human skeletal muscle biopsies from pre-cachectic and cachectic pancreatic cancer patients compared to control individuals (Ctr, n=41; pre-cachectic, n=32; cachectic, n=48; control vs cachexia q value=0.0022; pre-cachexia vs cachexia q value=0.0091;). Values are reported as fold change over control group**. e)** C2C12 myotubes diameter treated with 20% C26 CM for 24h. (n=5) **f)** mRNA levels of FKBP12 relative to GAPDH in the gastrocnemii of C26 tumor-bearing mice and relative control mice 11 days post cancer cells inoculation. (n=8) **g)** Immunoblot and densiometric quantification of FKBP12 in the quadriceps of control and C26 tumor-bearing mice. (n=3/4) **h)** Expression levels of FKBP12 in C2C12 myotubes treated for 48h with 20% C26 CM (n=6). **i)** Putative STAT3 binding site on *Fkbp12* promoter. **j-k)** Time course and immunoblot of phosphorylated STAT3 (Tyr705 – pSTAT3) and total STAT3 in C2C12 myotubes serum-starved for 2h and treated with pure C26 CM **(j)** (n=2) or IL-6 (100ng/mL) **(k)** or serum-free DMEM (CTR) at different timepoints. (n=2) **l-m)** ChIP-qPCR on a negative control region characterized by the absence of pSTAT3 and H3K27Ac in C2C12 myotubes treated with C26 CM (**l**) and *in vivo* skeletal muscles (**m**) compared to the control (each condition represents the pooling of 4 quadriceps). (n=2)

Data information: Statistical significance was tested with unpaired two-tailed student's t-test in graphs a, b, e-h; with Kruskal-Wallis test followed by Benjamini, Krieger and Yekutieli multiple comparison test in graphs c, d; with two-way ANOVA followed by Sidak's multiple comparison test in graphs l-m.

Supplementary Figure 2

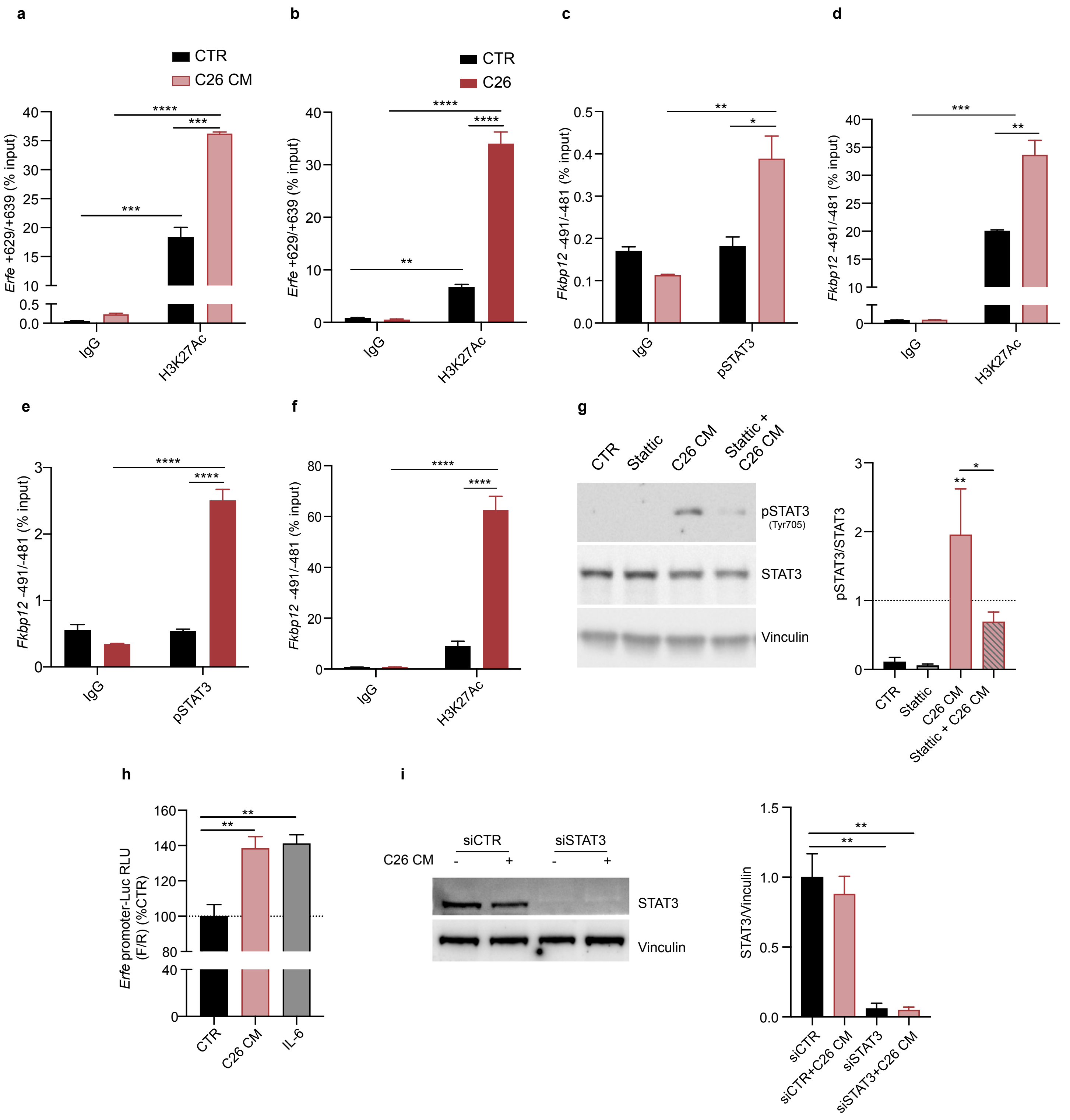

Figure S2: The BMP-scavenger erythroferrone (ERFE) is upregulated in cachexia through STAT3 activation. Related to Fig. 1.

a) ChIP-qPCR of acetylated H3K27 of *Erfe* promoter on TSS (+629/+639 bp) in C2C12 myotubes treated with C26 CM compared to control cells. (n=2) **b)** *In vivo* ChIP-qPCR of acetylated H3K27 of *Erfe* promoter on TSS (+629/+639 bp). ChIP-qPCR assay was performed by pooling 4 quadriceps per condition for control or C26 tumor-bearing mice. (n=2). **c-d)** *In vitro* ChIP-qPCR of pSTAT3 binding *Fkbp12* promoter on TSS (-491/-481) (**c**). The binding sites also carried an enrichment of the histone modification-H3K27ac as indication of open chromatin (**d**). ChIP-qPCR assay was performed on C2C12 myotubes serum-starved for 2h and treated for 30' with pure C26 CM. (n=2) **e-f)** *In vivo* ChIP-qPCR of pSTAT3 binding *Fkbp12* promoter on TSS (-491/-481) (**e**). The pSTAT3 binding sites also carried the histone modification-H3K27ac (**f**). ChIP-qPCR assay was performed by pooling 4 quadriceps per condition for control or C26 tumor-bearing mice. (n=2) **g)** Representative immunoblot of pSTAT3 (Tyr705) and relative densitometric quantification of C2C12 myotubes treated with Stattic 5uM and/or pure C26 CM for 30', previously serum-starved for 2h. (n=3) **h)** Luminescence signal (firefly/renilla) of NIH/3T3 cells transfected with *Erfe* promoter-Luc reporter vector and treated with 50% C26 CM or IL-6 (100ng/mL) overnight (n=4/6). **i)** Representative STAT3 immunoblot and relative densitometric quantification of NIH/3T3 cells transfected as in Fig. 1m (n=2).

Data information: Statistical significance was tested with unpaired two-tailed student's t-test in graph h; with two-way ANOVA followed by Sidak's multiple comparison test in graphs a-f, and with one-way ANOVA followed by Sidak's multiple comparison test in graph g, i.

Supplementary Figure 3

a

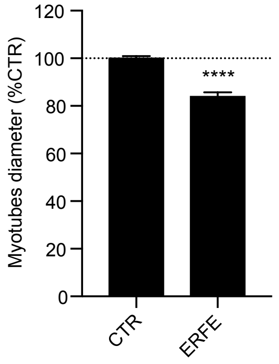

b

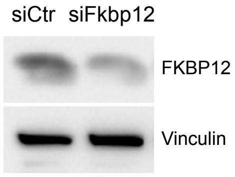

Figure S3: Muscle-specific knockdown of BMP inhibitors curbs muscle wasting in C26 tumor-bearing mice. Related to Fig. 2.

a) Quantification of C2C12 myotubes treated with murine recombinant ERFE (1ug/mL) for 24h. (n=5/7) **b)** Immunoblotting for *Fkbp12* knock-down in myotubes transfected with siRNA for *Fkbp12* or scramble sequence (siCtr) for 48h (n=2).

Data information: Statistical significance was tested with unpaired two-tailed student's t-test in graph a.

Supplementary Figure 4

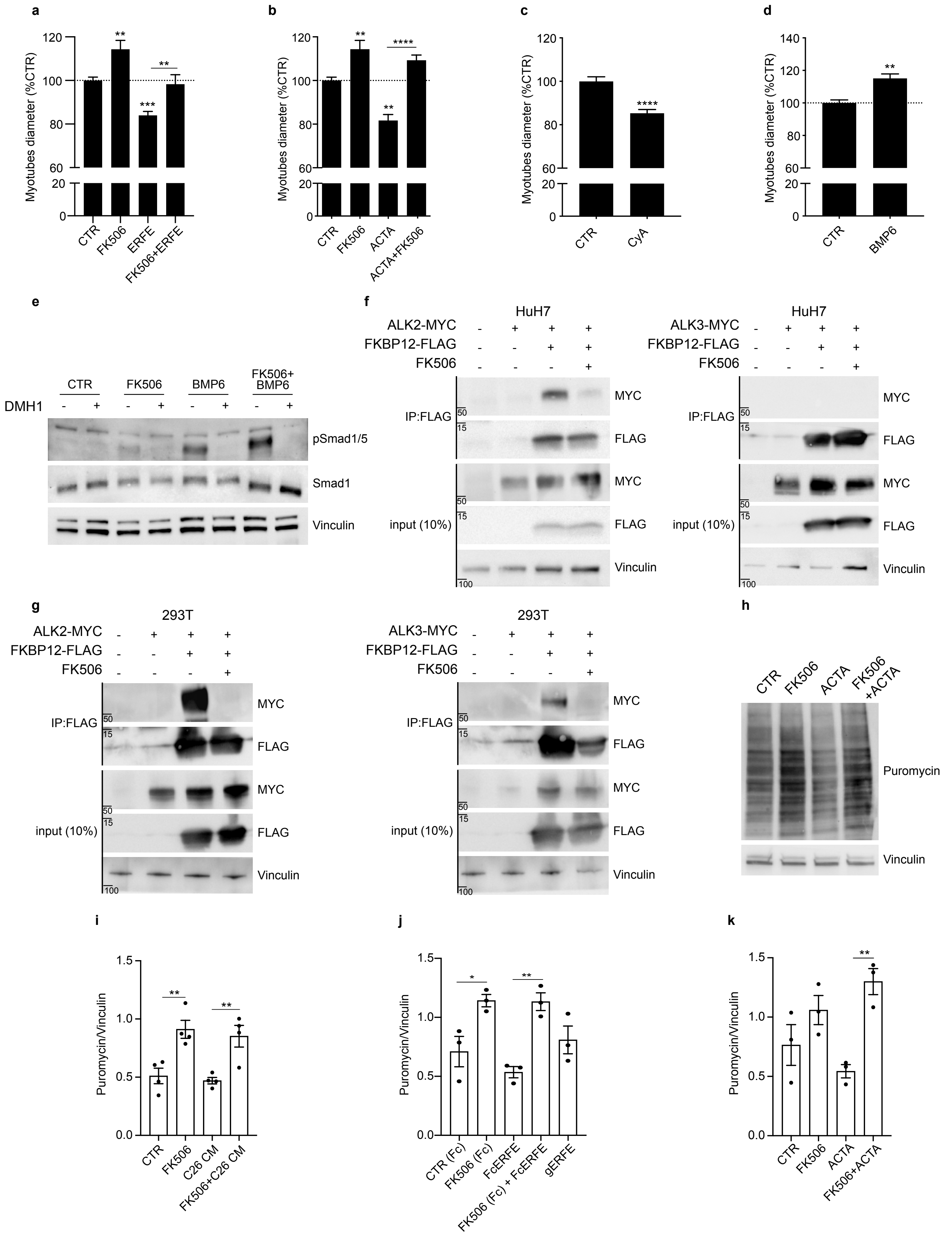

Figure S4: FK506 bypasses BMP-pathway inhibition by activating pSmad1/5/8 through FKBP12 binding. Related to Fig. 3.

a-d) Myotubes diameters quantified at 24h after treatment with FK506 (1ug/mL), ERFE (1ug/mL) (**a**), Activin A (ACTA) (1ng/mL) (**b**), cyclosporine A (CyA) (1uM) (**c**), or BMP6 (1ng/mL) (**d**) in differentiation medium. (n=3/5) **e)** Immunoblot for pSmad1/5/8 and Smad1 of C2C12 myotubes serum-starved for 2h and treated with FK506 (1ug/mL), BMP6 (30ng/mL) with or without DMH1 (500nM) for 1h in serum-free DMEM. (n=3) **f-g)** HuH7 (**f**) and HEK-293T (**g**) cells were transfected with FKBP12-FLAG and ALK2-MYC or ALK3-MYC constructs for 48h. Afterwards, cells were switched to low serum medium (DMEM 2%FBS) for 3h and treated for 1h with FK506 (10ug/mL for HEK-293T and 1ug/mL for HuH7). Protein lysates were then immunoprecipitated using FLAG-tagged resin and immunoblotting was performed to detect the interaction between FKBP12 and ALK2 or ALK3. (n=4) **h)** *In vitro* SuNSET assay performed in C2C12 myotubes treated with FK506 (1ug/mL) and Activin A (ACTA) (1ng/mL) for 24h in differentiation medium. For the last 4h of treatment, puromycin (1uM) was added to the cells. (n=3) **i-k)** Densiometric quantifications of *in vitro* SuNSET assays performed in C2C12 myotubes treated with FK506, C26 CM (**i**), FcERFE (**j**) or ACTA (**k**). (n=3/4)

Data information: Statistical significance was tested with one-way ANOVA followed by Sidak's multiple comparison test in graph a, b, i-k; with unpaired two-tailed student's t-test in graphs c, d.

Supplementary Figure 5

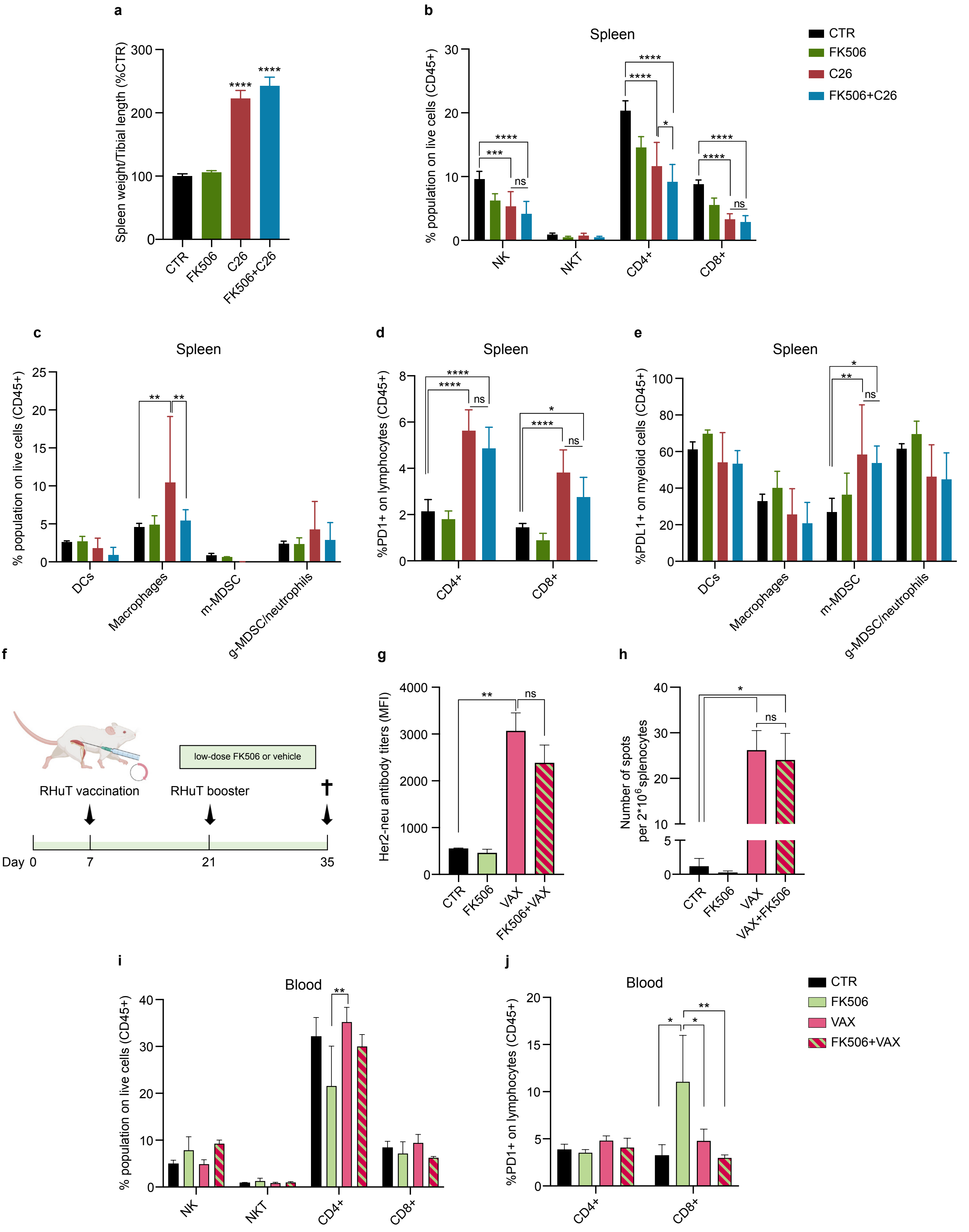

Figure S5: FK506 effect on the immune system *in vivo*. Related to Fig. 4.

a) Spleen weights normalized for tibial length in C26 tumor-bearing mice treated with FK506 and relative control groups. (n=11) **b-c)** Percentages of lymphoid (**b**) and myeloid (**c**) populations in the spleen of tumor and non-tumor bearing mice, treated or not with FK506. (n=4/6) **d-e)** Percentages of PD-1+ lymphocytes (**d**) and PD-L1+ myelocytes (**e**) in the spleens. (n=4/6) **f)** Experimental design of long-term low-dose FK506 in non-tumor bearing mice immunized with RHuT vaccine. BALB/c mice were pre-treated for 1 week with FK506 (0.02mg/Kg) before being vaccinated with RHuT plasmid or empty vector. Vaccination was repeated after 2 weeks, and mice were euthanized 35 days after the beginning of the experiment. All mice were daily treated with FK506 or vehicle through oral gavage. **g)** Titers of anti-Her2-neu antibodies (mean fluorescence intensity) in the sera of mice vaccinated (VAX) or not and treated with FK506 or vehicle. (n=3/5) **h)** ELISpot assay performed on splenocytes isolated from vaccinated and unvaccinated mice to measure IFN-γ-secreting cytotoxic T cells activated by the immunodominant peptide for ErbB2. (n=3/4) **i)** Amounts of lymphoid populations in the blood of vaccinated (VAX) or unvaccinated mice treated with chronic low-dose FK506 or vehicle. (n=3/5) **j)** Percentages of PD-1+ lymphocytes in the blood of the experimental groups of Fig. S5g. (n=3/5)

Data information: Statistical significance was tested with one-way ANOVA followed by Sidak's multiple comparison test in graph a, g, h; with two-way ANOVA in graphs b-e, i, j.

Supplementary Figure 6

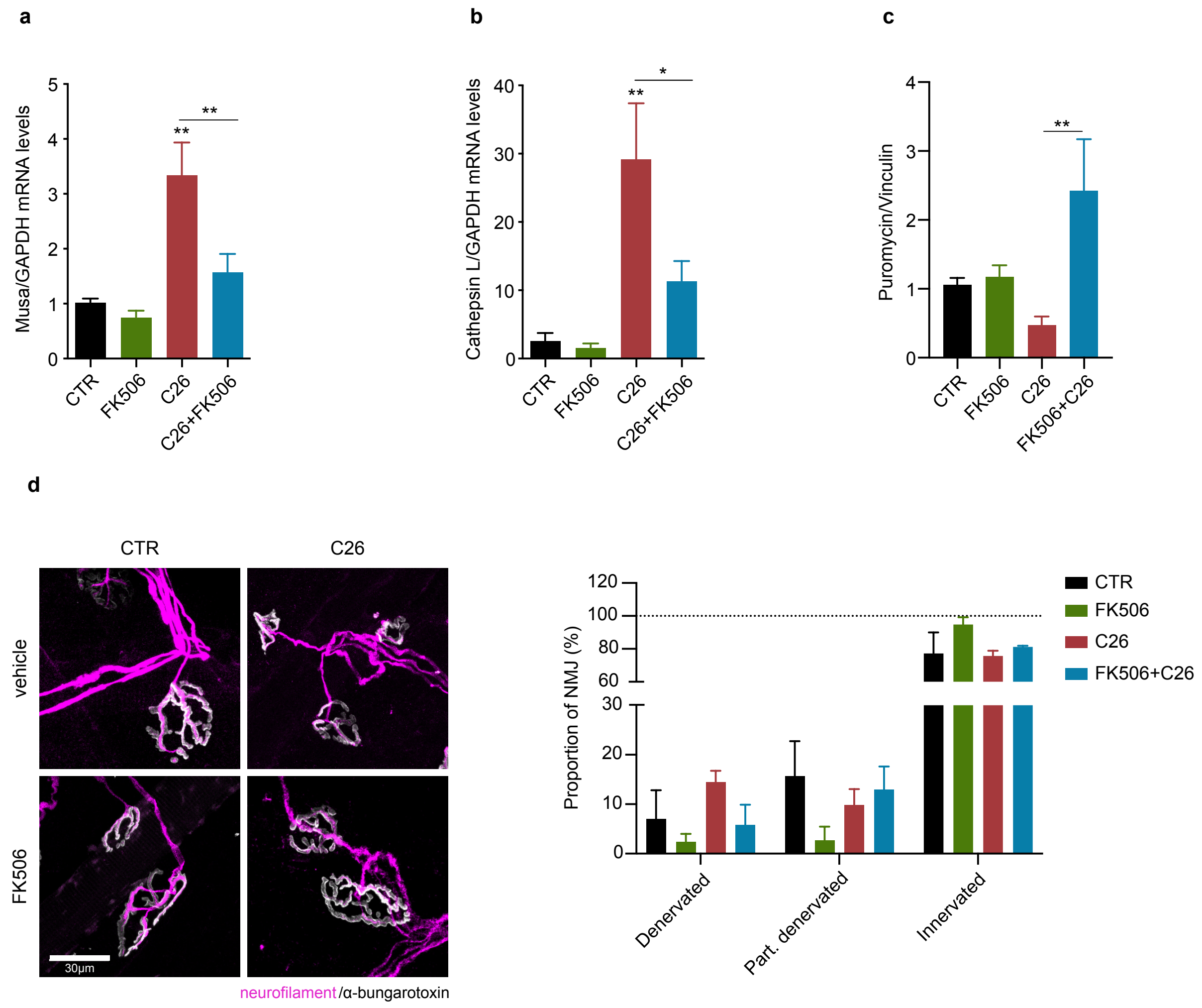

Figure S6: FK506 protects from cachexia, prevents the degeneration of neuromuscular junction morphology, and preserves muscle strength in C26 tumor-bearing mice. Related to Fig. 5 and Fig. 6.

a-b) Expression levels of the atrogenes Musa (**a**) and Cathepsin L (**b**) in the gastrocnemii of C26 and control mice treated with low-dose FK506 or vehicle. (n=4/6) **c)** Densitometric quantification of the *in vivo* SuNSET assay reported in Fig. 5h. (n=3/4). **d)** Representative immunofluorescence pictures and relative quantification of EDL muscles stained for α-bungarotoxin and anti-Neurofilament antibody to detect NMJs innervation. The quantification of denervated NMJs is expressed as percentage of the total number of NMJs quantified (60 to 90 NMJs per mouse, n=3 per group).

Data information: Statistical significance was tested with one-way ANOVA followed by Sidak's multiple comparison test in all graphs.

Video S1) FK506 protects from cachexia in C26 tumor-bearing mice. Related to Fig. 5.

C26-tumor bearing mice treated with vehicle (left) or FK506 (right) at day 11 post-C26 injection. Related to Fig. 5, 6 and Fig. S6.

Table S1

Gene	Species	Forward	Reverse
Atrogin1	Mouse	ATGCACACTGGTGCAGAGAG	TGTAAGCACACAGGCAGGTC
Murf1	Mouse	GTGTGAGGTGCCTACTTGCTC	GCTCAGTCTTCTGTCCTTGGA
Musa1	Mouse	TCGTGGAATGGTAATCTTGC	CCTCCCGTTTCTCTATCACG
Cathepsin L	Mouse	GTGGACTGTTCTCACGCTC	TCCGTCCTTCGCTTCATAGG
FKBP12	Mouse	ATGGGAGTGCAGGTGGAG	TCTTTCCATCTTCAAGCATCC
ERFE	Mouse	TGCTTGGATGCTGTTCGTCAA	CAGATGGGATAAAGGGGCCTG
GAPDH	Mouse	AGGTCGGTGTGAACGGATTTG	TGTAGACCATGTAGTTGAGGTCA
BMP6	Mouse	GGGATGGCAGGACTGGATCA	ATGGTTTGGGGACGTACTCG
BMP2	Mouse	CATCACGAAGAAGCCGTGGA	TGAGAAACTCGTCACTGGGG
BMP4	Mouse	TTCCTGGTAACCGAATGCTGA	CCTGAATCTCGGCGACTTTTT
Chrne	Mouse	GTGTCTGGATTGGCATTGACT	ACACCTGCAAAATCGTCCTTG
ERFE	Human	GGCCAAGAAGCTGAAGTTCGG	CTGCCGCACCGCACCTTTC
Beta Actin	Human	GGGAAATCGTGCGTGACA	GGACTCCATGCCCAGGA
ChIP-qPCR Erfe promoter	Mouse	GGGTGGATTGGTAGCCTTCA	TGCCTTGCAGAGGGAGGTAT
ChIP-qPCR Fkbp12 promoter	Mouse	ATTCGTGGAAGAGTGGGCTT	TGCCCTGTAAGGACGATAGC
ChIP-qPCR Negative Ctr	Mouse	TGGCCCCATAGGCTCATCTA	GGCCTGGCATGAGAGACTTT

Table S1: RT-qPCR SYBR primers and ChIP-qPCR primers. Related to STAR Methods.
