# Supplementary material for: FK506 bypasses the effect of erythroferrone in cancer cachexia skeletal muscle atrophy
Source: Cell Rep Med. 2023 Dec 4;4(12):101306. doi: 10.1016/j.xcrm.2023.101306 (PMC10772350; doi:10.1016/j.xcrm.2023.101306)
Supplement: Document S2. Article plus supplemental information [file mmc3.pdf]

Cell Reports Medicine

Article

FK506 bypasses the effect of erythroferrone in cancer cachexia skeletal muscle atrophy

Graphical abstract

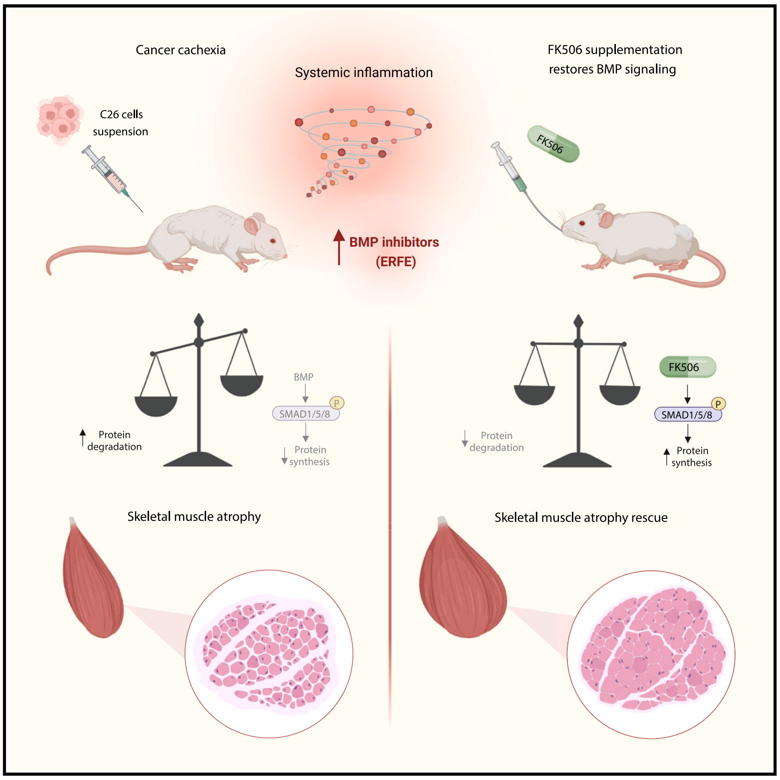

Highlights

- The BMP-scavenger erythroferrone is upregulated in cachectic skeletal muscles
- The BMP signaling inhibitors erythroferrone and FKBP12 promote muscle atrophy
- FK506 activates BMP signaling by targeting FKBP12
- FK506 prevents muscle wasting and increases protein synthesis in cancer cachexia

Authors

Erica Mina, Elisabeth Wyart, Roberta Sartori, ..., Léon Kautz, Laura Silvestri, Paolo E. Porporato

Correspondence

paolo.porporato@unito.it

In brief

Mina et al. explore the effects of FK506 treatment in skeletal muscle atrophy during cancer cachexia. This condition is characterized by reduced BMP signaling, leading to increased protein degradation and, therefore, muscle atrophy. FK506 re-activates this pathway rescuing protein synthesis, muscle force, and neuromuscular junction perturbations in cachexia.

Mina et al., 2023, Cell Reports Medicine *4*, 101306
December 19, 2023 © 2023 The Author(s).
https://doi.org/10.1016/j.xcrm.2023.101306

CellPress

Cell Reports Medicine

CellPress
OPEN ACCESS

Article

FK506 bypasses the effect of erythroferrone in cancer cachexia skeletal muscle atrophy

Erica Mina,[1] Elisabeth Wyart,[1] Roberta Sartori,[2,3] Elia Angelino,[1,4] Ivan Zaggia,[1] Valentina Rausch,[1] Mara Maldotti,[5,6] Alessia Pagani,[7] Myriam Y. Hsu,[1,8] Alberto Friziero,[9,10] Cosimo Sperti,[11] Alessio Menga,[1] Andrea Graziani,[1,4] Emilio Hirsch,[1] Salvatore Oliviero,[5,6] Marco Sandri,[2,3] Laura Conti,[1] Léon Kautz,[12] Laura Silvestri,[7,13] and Paolo E. Porporato[1,14,*]

[1]Department of Molecular Biotechnology and Health Sciences, Molecular Biotechnology Center "Guido Tarone", University of Torino, 10126 Torino, Italy
[2]Department of Biomedical Sciences, University of Padova, Padova, Italy
[3]VIMM: Veneto Institute of Molecular Medicine, Padova, Italy
[4]Department of Translational Medicine, University of Piemonte Orientale, 28100 Novara, Italy
[5]Department of Life Sciences and Systems Biology, Molecular Biotechnology Center "Guido Tarone", University of Torino, 10126 Torino, Italy
[6]Italian Institute for Genomic Medicine (IIGM), Sp142 Km 3.95, 10060 Candiolo, Torino, Italy
[7]Division of Genetics and Cell Biology, IRCCS Ospedale San Raffaele, Milan, Italy
[8]Division of Cell Fate Dynamics and Therapeutics, Department of Biosystems Science, Institute for Life and Medical Sciences (LiMe), Kyoto University, Kyoto, Japan
[9]Department of Surgery, Oncology and Gastroenterology, University of Padova, Padova, Italy
[10]General Surgery 1, Padova University Hospital, Padova, Italy
[11]General Surgery 2, Hepato-Pancreato-Biliary Surgery and Liver Transplantation Unit, Padova University Hospital, Padova, Italy
[12]IRSD, Université de Toulouse, INSERM, INRAE, ENVT, University Toulouse III - Paul Sabatier (UPS), Toulouse, France
[13]Vita Salute San Raffaele University, Milan, Italy
[14]Lead contact
*Correspondence: paolo.porporato@unito.it
https://doi.org/10.1016/j.xcrm.2023.101306

SUMMARY

Skeletal muscle atrophy is a hallmark of cachexia, a wasting condition typical of chronic pathologies, that still represents an unmet medical need. Bone morphogenetic protein (BMP)-Smad1/5/8 signaling alterations are emerging drivers of muscle catabolism, hence, characterizing these perturbations is pivotal to develop therapeutic approaches. We identified two promoters of "BMP resistance" in cancer cachexia, specifically the BMP scavenger erythroferrone (ERFE) and the intracellular inhibitor FKBP12. ERFE is upregulated in cachectic cancer patients' muscle biopsies and in murine cachexia models, where its expression is driven by STAT3. Moreover, the knock down of *Erfe* or *Fkbp12* reduces muscle wasting in cachectic mice. To bypass the BMP resistance mediated by ERFE and release the brake on the signaling, we targeted FKBP12 with low-dose FK506. FK506 restores BMP-Smad1/5/8 signaling, rescuing myotube atrophy by inducing protein synthesis. In cachectic tumor-bearing mice, FK506 prevents muscle and body weight loss and protects from neuromuscular junction alteration, suggesting therapeutic potential for targeting the ERFE-FKBP12 axis.

INTRODUCTION

Cachexia is a progressive debilitating syndrome that can be associated with several systemic pathologies, such as cancer and sepsis, and it is characterized by severe wasting of skeletal muscle and adipose tissue.[1] Cancer cachexia is correlated with a worse prognosis and therapy responses, accounting for at least 30% of deaths in advanced cancer patients. Despite the high prevalence of this disease, the poor molecular understanding of the pathology makes cachexia still an unmet medical need.[2] The relevance of targeting skeletal muscle atrophy is supported by the fact that not only it is a predictor of poor clinical outcomes[3] and reduction of quality of life,[4] but its prevention prolongs survival in cachectic tumor-bearing mice, independent of tumor growth, fat loss, and inflammation.[5]

Skeletal muscle atrophy is the result of unbalanced protein and organelles catabolism, coupled with reduced anabolic processes and metabolic rewiring. Indeed, mitochondrial dysfunction, zinc overload and iron deficiency have been associated with body weight loss in cachexia,[6–8] with mitochondrial alterations preceding the onset of weight loss.[9,10]

From a molecular standpoint, muscle mass maintenance is regulated by different signaling pathways that tune protein synthesis and degradation, notably the transforming growth factor β (TGFβ) receptor superfamily,[11–15] ubiquitin-proteasome axis,[16–18] calcium-dependent proteolysis,[19] and autophagy cascades.[20]

 © 2023 The Author(s).
This is an open access article under the CC BY license (http://creativecommons.org/licenses/by/4.0/).

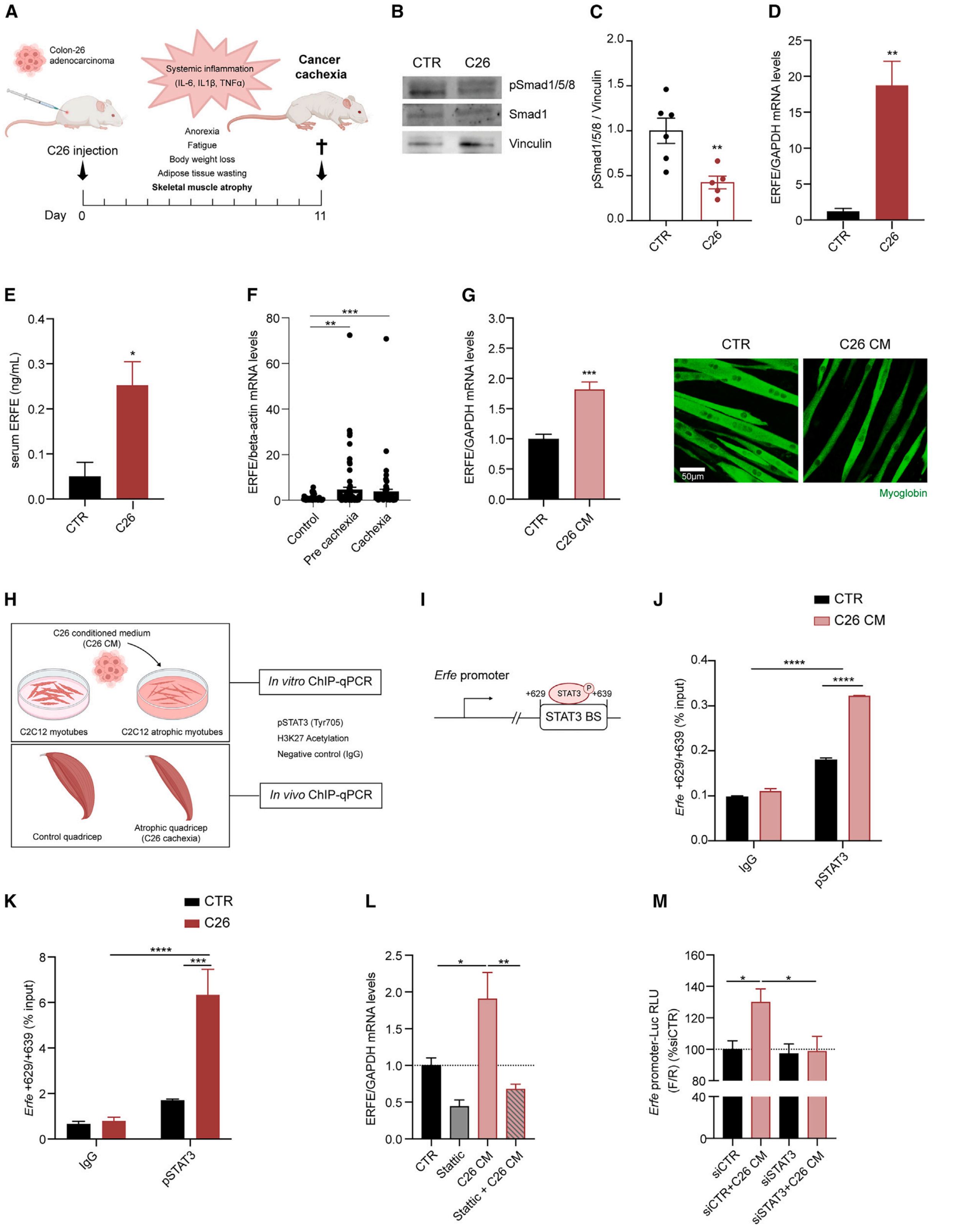

(legend on next page)

Ligands as activins, myostatin, or TGFβ, preferentially bind activin receptors (ActRIIB, ActRIIA, TGFβRII, and ALK-4, ALK-7, and ALK-5) triggering Smad2/3 phosphorylation and inducing muscle atrophy and cachexia.[21–25]

On the other hand, bone morphogenetic proteins (BMPs) are positive regulators of muscle mass, that by binding BMP receptors (BMPRII, ActRIIB, ActRIIA, and ALK1, ALK2, ALK3, and ALK6), activate biosynthetic pathways through Smad1/5/8 phosphorylation,[12,13] and inhibit proteolysis by downregulating the expression of atrophy-associated E3 ubiquitin ligases ("atrogenes").[11–13]

Moreover, BMP-Smad1/5/8 axis has also been shown to regulate neuromuscular junction (NMJ) formation and remodeling in models of *Drosophila*,[26,27] and Noggin-mediated BMP inhibition induces skeletal muscle fibers denervation in cancer cachexia.[14]

BMP signaling is a complex network that can be regulated at different levels. Since we previously reported that iron metabolism is dysregulated in cachexia,[7] we explored the impact on skeletal muscle of different BMP inhibitors involved in the iron field. For instance, erythroferrone (ERFE) is an erythroid regulator of iron metabolism secreted in the circulation mainly by erythroid precursors[28] that scavenges BMP6 homodimers[29] and BMP6-BMP2 heterodimers[30] downregulating Smad1/5/8 phosphorylation in the liver.[30,31] Interestingly, ERFE was found to be also expressed in the skeletal muscle (named as myonectin[32]), but besides its extensively studied effects on the liver, its role on BMP signaling in the skeletal muscle has not been elucidated.

A further layer of BMP-Smad pathway regulation in the liver is represented by FKBP12 (FK506 binding protein 12),[33,34] an immunophilin that limits and prevents the uncontrolled activation of BMP signaling,[35] by binding the cytoplasmic domain of type I BMPR (BMPR-I).[36]

Given that BMP-Smad1/5/8 axis counteracts the catabolic TGFβ-Smad2/3 pathway in the skeletal muscle, we evaluated the role of ERFE and FKBP12 in regulating muscle mass in wasting conditions.

In our study, we demonstrated that the main inflammatory pathway associated to cachexia (IL-6-STAT3 axis) upregulates the BMP signaling inhibitor *Erfe* in atrophic skeletal muscles, leading to a condition of BMP resistance that can contribute to the catabolic effects of the hyper-active activin/TGFβ-Smad2/3 signaling.

Furthermore, we propose a therapeutic approach to bypass ERFE-mediated BMP resistance by displacing the inhibitor FKBP12 from the BMPR-I with FK506 (tacrolimus), in models of cancer cachexia *in vitro* and *in vivo*.

RESULTS

The BMP scavenger ERFE is upregulated in cachexia through STAT3 activation

BMP inhibitors such as Noggin[14] and myostatin[21] are upregulated both in mouse and human cancer cachexia.[14,21] The BMP-Smad1/5/8 axis in the liver is downregulated by scavengers like ERFE, an erythroid regulator of iron metabolism[28] that, once secreted in the circulation, chelates BMP ligands.[30,31] However, whether this regulator plays a role not only in the liver[14] but also in muscle wasting and cancer cachexia is not known.

Mice carrying colon-26 (C26) adenocarcinoma developed severe cancer cachexia within 11 days and in their atrophic muscles phosphorylated Smad1/5/8 were downregulated as previously described[14] (Figures 1A–1C and S1A). Intriguingly, we found that C26 tumor-bearing mice upregulated the BMP scavenger *Erfe* as mRNA levels (Figure 1D) in their atrophic skeletal muscles, and as protein levels in the serum (Figure 1E). While the reduction of the BMP-Smad1/5/8 axis is in line with increased levels of *Erfe*, we do not observe a decreased expression of BMP ligands, such as BMP2, BMP4, and BMP6, in the skeletal muscle of C26 tumor-bearing mice (Figure S1B).

Figure 1. The BMP scavenger ERFE is upregulated in cachexia through STAT3 activation

(A) Schematic representation of the C26 cancer cachexia model where mice injected with C26 cells develop severe cachexia within 11 days.

(B) Immunoblot of phosphorylated Smad1/5/8 (pSmad1/5/8) and vinculin in the quadriceps of control and C26 tumor-bearing mice (n = 5/6).

(C) Densiometric quantification of the immunoblot of pSmad1/5/8 and Smad1 in the quadriceps of control and C26 tumor-bearing mice (n = 5/6).

(D) mRNA levels of ERFE relative to GAPDH in the gastrocnemii of C26 tumor-bearing mice and relative control mice 11 days post cancer cells inoculation (n = 4/5). GAPDH, glyceraldehyde-3-phosphate dehydrogenase.

(E) ERFE protein concentration detected by ELISA in the serum of C26 and control mice (n = 4).

(F) Expression levels of ERFE relative to beta-actin in human skeletal muscle biopsies from pre-cachectic and cachectic colorectal and pancreatic cancer patients compared to control individuals (control, n = 41; pre-cachectic, n = 77; cachectic, n = 63). Values are reported as fold change over control group (control vs. pre-cachexia q value = 0.0021; control vs. cachexia q value = 0.0007).

(G) Expression levels of *Erfe* in C2C12 myotubes treated for 48h with 20% C26 CM (n = 6) and representative immunofluorescence of atrophic C2C12 myotubes treated with C26 CM and stained for myoglobin.

(H) Experimental design of the ChIP-qPCR assay performed on C2C12 myotubes treated with C26 CM or atrophic skeletal muscles of C26 tumor-bearing mice at day 11.

(I) STAT3 predicted binding sites on *Erfe* promoter.

(J) ChIP-qPCR of pSTAT3 binding *Erfe* promoter on TSS (+629/+639 bp) in C2C12 myotubes treated with C26 CM compared with control cells (n = 2).

(K) *In vivo* ChIP-qPCR of pSTAT3 binding *Erfe* promoter on TSS (+629/+639 bp). ChIP-qPCR assay was performed by pooling 4 quadriceps per condition for control or C26 tumor-bearing mice (n = 2).

(L) Expression levels of *Erfe* on C2C12 myotubes treated with Stattic 5 μM and/or 50% C26 CM for 6 h (n = 3).

(M) Luminescence (firefly/renilla) of NIH/3T3 transfected with *Erfe* promoter-Luc reporter vector and knocked down for STAT3 (siSTAT3). Cells were treated overnight with 50% C26 CM prior to performing the assay (n = 2). See also Figures S1 and S2. Data information: Statistical significance was tested with unpaired two-tailed Student's *t*-test in (C)–(E) and (G); with non-parametric Krustal-Wallis test followed by Benjamini, Krieger, and Yekutieli multiple comparison tests in (F) and with two-way ANOVA followed by Sidak's multiple comparison test in (J) and (K) and with one-way ANOVA followed by Sidak's multiple comparison test in (L) and (M).

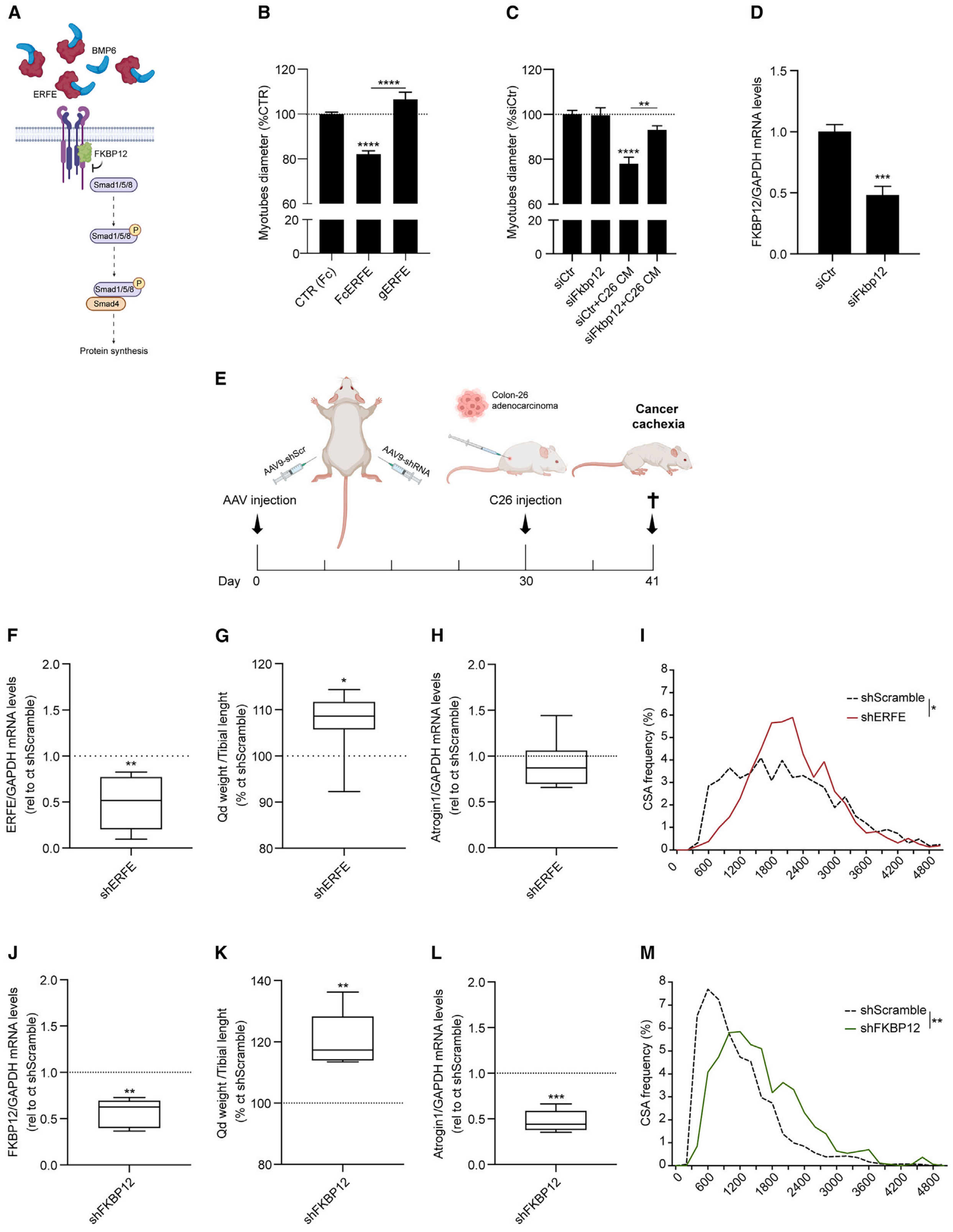

(legend on next page)

We then analyzed the expression levels of *ERFE* in human samples from oncology patients. We found that *ERFE* expression is significantly higher in skeletal muscle biopsies from pre-cachectic (body-weight loss in 6 months, or <2% in patients with low muscle mass, or a body mass index [BMI] of <20) and cachectic (body-weight loss in 6 months, or >2% in patients with low muscle mass, or a BMI of <20) patients with colorectal or pancreatic cancer, than in cancer-free controls (Figures 1F, S1C, and S1D). In agreement, atrophic myotubes obtained by treating C2C12 with C26 conditioned medium (CM), displayed significant higher transcripts levels of the BMP inhibitor *Erfe* (Figures 1G and S1E).

To explore potential targets for BMP-signaling restoration, we analyzed in our models the expression levels of the immunophilin FKBP12 as it docks BMPR-I to limit their downstream signaling in the absence of ligands.[35,36] The mRNA levels of the intracellular inhibitor *Fkbp12* were significantly upregulated in atrophic skeletal muscles of C26 tumor-bearing mice (Figure S1F), accompanied by a trend in increased protein levels (Figure S1G) and transcript in C2C12-derived atrophic myotubes (Figure S1H).

Subsequently, we explored which molecular mechanism could trigger the transcriptional up-regulation of the BMP inhibitors *Erfe* and *Fkbp12* in cachexia by performing chromatin immunoprecipitation (ChIP-qPCR) assay on atrophic C2C12 myotubes treated with C26 CM and on cachectic skeletal muscles of C26 tumor-bearing mice (Figure 1H). JASPAR TEFBS enrichment analysis[37] of *Erfe* and *Fkbp12* promoter sequences (±1,000 bp from the transcription start site [TSS]), showed putative top-ranked predicted binding sites of STAT3 downstream *Erfe* TSS (+629/+639 bp) and upstream *Fkbp12* TSS (−491/-481) (Figures 1I and S1I). Indeed, the IL-6-STAT3 axis is a well-known trigger of muscle wasting in tumor-bearing mice.[38–41] In agreement, in C2C12 myotubes, C26 CM, as well as IL-6, strongly induced phosphorylation of STAT3 at Tyr705 (pSTAT3) (Figures S1J and S1K).

We, therefore, evaluated whether pSTAT3 could be involved in *Erfe* upregulation and *Fkbp12* expression in cachexia. We found that pSTAT3 significantly bound *Erfe* promoter in atrophic C2C12 myotubes treated with C26 CM (Figures 1J and S1L). Furthermore, this promoter region was proven to be transcriptionally active because it carried the histone modification-H3K27ac, a known marker for chromatin accessibility[42] (Figure S2A). In a similar way, pSTAT3 strongly bound *Erfe* promoter in atrophic muscles (Figures 1K, S1M, and S2B), indicating that activated STAT3 can be responsible for the upregulation of the BMP scavenger *Erfe* in cachexia.

Our ChIP-qPCR demonstrated that pSTAT3 can also bind the active *Fkbp12* promoter in atrophic myotubes (Figures S2C and S2D) and in cachectic C26 muscles (Figures S2E and S2F), suggesting that activated STAT3 is potentially involved in the regulation of BMP-signaling inhibitors.

Since ChIP-qPCR assays only provide information on the binding of transcription factors to specific sequences, we evaluated whether STAT3 is required for *Erfe* upregulation. By treating atrophic C2C12 myotubes with Stattic, an inhibitor of STAT3 phosphorylation and nuclear translocation,[43] we blunted the up-regulation of *Erfe* mediated by C26 CM (Figures 1L and S2G). Moreover, to evaluate transcriptional activation of the target gene, we generated a luciferase reporter under the control of the murine *Erfe* promoter.

As expected, treatment with C26 CM or IL-6, which strongly induce pSTAT3, significantly triggers luciferase signal in transfected cells (Figure S2H). Consistently, the increase of luciferase signal induced by C26 CM is blunted when cells are knocked down for STAT3 (Figures 1M and S2I), further proving the crucial role of STAT3 in driving *Erfe* upregulation.

These results indicate that pSTAT3 not only binds *Erfe* promoter in cachexia, but its activation is also required for *Erfe* upregulation.

Muscle-specific knockdown of BMP inhibitors curbs muscle wasting in C26 tumor-bearing mice

To understand the biological role of the BMP-signaling inhibitors ERFE and FKBP12 in cachexia (Figure 2A), we treated C2C12-derived myotubes with recombinant ERFE, using the truncated-inactive form (gERFE) as a negative control. ERFE was sufficient to induce atrophy *in vitro* (Figures 2B and S3A), while gERFE did not, thus suggesting that the functional N-terminal

Figure 2. Muscle-specific knockdown of BMP inhibitors curbs muscle wasting in C26 tumor-bearing mice

(A) Schematic representation of the pathway. The BMP scavenger ERFE is upregulated in cachexia and with the intracellular inhibitor FKBP12 contributes to the downregulation of pSmad1/5/8 in atrophic muscles.

(B) Quantification of myotubes diameters after 24 h treatment with recombinant Fc-ERFE (1 μg/mL), inactive globular Fc-gERFE (1 μg/mL), and the relative Fc-CTR (1 μg/mL) (n = 3/4).

(C) Myotubes diameters transfected with siRNA for *Fkbp12* or scramble sequence (siCtr) for 48 h and treated for 24 h with 10% C26 CM (n = 3/4).

(D) Expression levels in C2C12 myotubes knocked-down for FKBP12 with siRNA (n = 4/6).

(E) Experimental design of muscle-specific AAV9 delivery in the skeletal muscle of C26 tumor-bearing mice. AAV9-shRNA (10^{11} vp) viral particles were injected in the skeletal muscles of BALB/c mice, using the contralateral limb as control (AAV9-shScr). One month after AAV injection, BALB/c mice were subcutaneously inoculated with 750,000 C26 cancer cells. The mice were euthanized 11 days after C26 injection.

(F) mRNA levels of ERFE in the quadriceps of AAV9-shERFE-injected mice vs. the contralateral AAV9-shScr limb (n = 7).

(G) Quadriceps weight normalized for tibial length compared with the contralateral limb (n = 7).

(H) Atrogin1 expression in the quadriceps analyzed in Figures 2F and 2G (n = 7).

(I) CSA distribution of AAV9-shERFE-injected mice (n = 3).

(J) mRNA levels of FKBP12 in the quadriceps of AAV9-shFKBP12-injected mice vs. the contralateral AAV9-shScramble limb (n = 7).

(K) Quadriceps weight normalized for tibial length compared with the contralateral limb (n = 7).

(L) Atrogin1 expression in the quadriceps analyzed in Figures 2J and 2K.

(M) CSA distribution of AAV9-shFKBP12-injected mice (n = 3). See also Figure S3. Data information: Statistical significance was tested with unpaired two-tailed Student's *t*-test in (D); with one-way ANOVA followed by Sidak's multiple comparison test in (B) and (C). One-sample *t*-test was performed in (F)–(H) and (J)–(L), while two-way ANOVA followed by Sidak's multiple comparison test in (I) and (M).

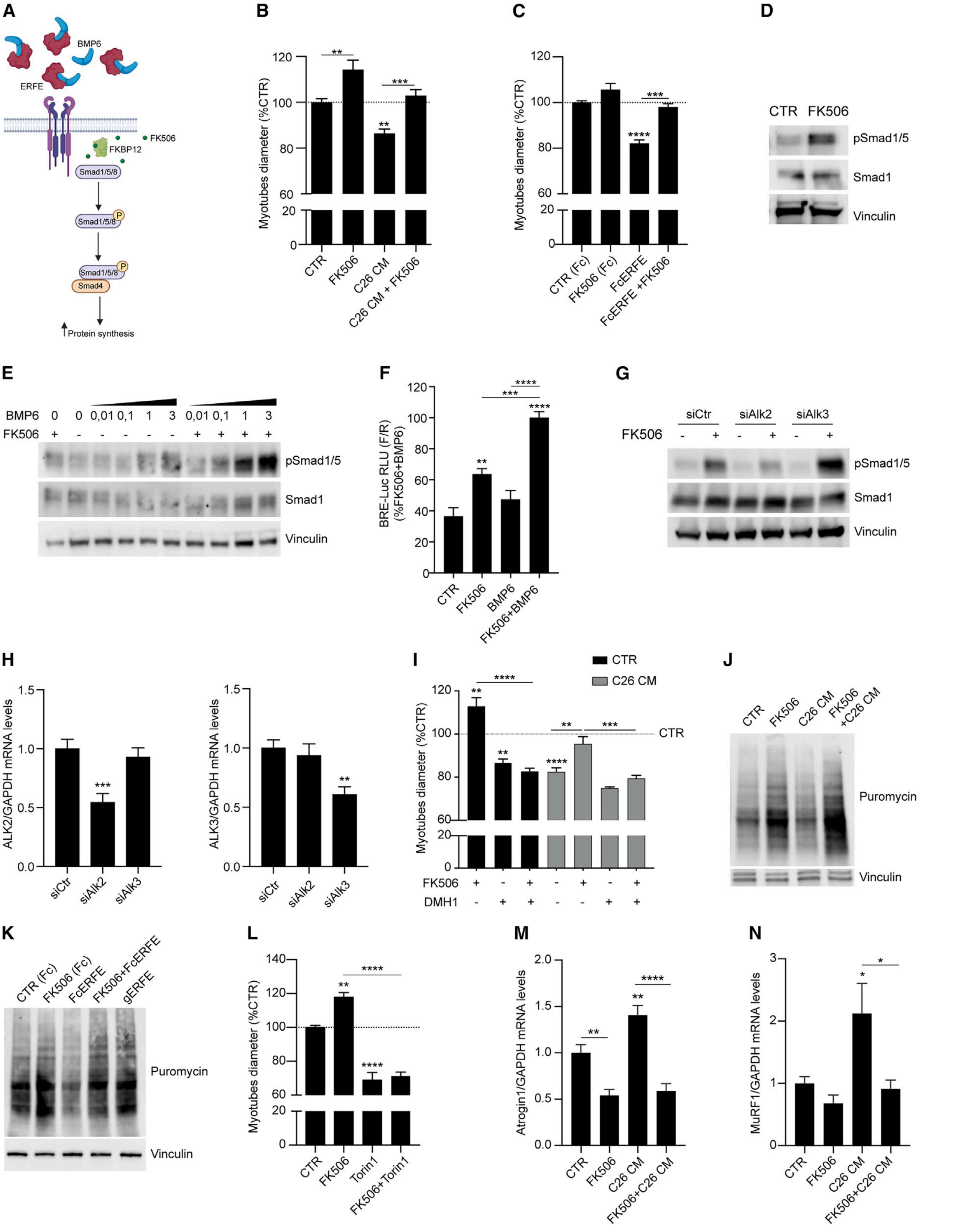

(legend on next page)

domain, known to downregulate the BMP-Smad signaling in the liver,[44] is biologically active in the skeletal muscle.

While the supplementation of the extracellular inhibitor ERFE induced myotube atrophy, the downregulation of the intracellular inhibitor *Fkbp12* by small interfering RNA (siRNA) was sufficient to rescue C26 CM-induced myotube atrophy (Figures 2C, 2D, and S3B).

We next explored *in vivo* whether ERFE or FKBP12 downregulation was sufficient to protect from muscle wasting in cachectic C26 tumor-bearing mice. We delivered adeno-associated viral particles with muscle tropism (AAV9) carrying short hairpin RNA (shRNA) targeting either *Erfe* (AAV9-sh*Erfe*) or *Fkbp12* (AAV9-sh*Fkbp12*) in one hindlimb of BALB/c mice and control viral particles (AAV9-shScramble carrying a scrambled sequence) in the contralateral limb. After inducing cachexia by injecting C26 cancer cells (Figure 2E), we found that *Erfe* knockdown (Figure 2F) in the quadricep was sufficient to reduce the weight loss detected in their contralateral muscle (Figure 2G) and this was accompanied by decreased Atrogin1 expression, a marker of the atrophic process (Figure 2H). These muscles also presented increased cross-sectional area (CSA) (Figure 2I), indicating that ERFE downregulation in the muscle is sufficient to curb the wasting process.

Following the same approach, we evaluated whether muscle-specific FKBP12 knockdown had a similar effect of AAV9-sh*Erfe* on muscle atrophy in C26-tumor bearing mice. *Fkbp12* downregulation (Figure 2J) potently increased quadriceps weight and significantly mitigated Atrogin1 levels in C26 tumor-bearing mice (Figures 2K and 2L), as well as displayed higher number of bigger fibers (Figure 2M) with respect to their contralateral limbs. Therefore, these results encouraged us to test whether FKBP12 can represent a valuable therapeutic target, restoring BMP signaling and counteracting cachexia.

FK506 bypasses BMP-pathway inhibition by activating pSmad1/5/8 through FKBP12 binding

Since *Fkbp12* knockdown protected from C26 cancer cells-induced muscle atrophy (Figures 2C and 2J–2M) and that BMP signaling is decreased in cancer-related skeletal muscle atrophy[14] (Figure 1B), we decided to bypass the inhibition of the BMP signaling using a pharmacological approach (Figure 3A). FK506 is a drug clinically used as an immunosuppressant that complexes with FKBP12 to inhibit calcineurin, thereby suppressing T cell proliferation.[45,46] Nevertheless, a non-immunosuppressive low dose of FK506 increased BMP-Smad1/5/8 signaling in hepatocytes[34] and in mouse models of chronic kidney, cardiac, and liver disease.[47]

Therefore, we treated C2C12 myotubes with FK506 to assess its effect on atrophic stimuli. FK506 treatment induced significant hypertrophy in myotubes and completely prevented the atrophy induced by C26 CM (Figure 3B) and recombinant ERFE (Figures 3C and S4A). Moreover, FK506 rescued the atrophy induced by activin A (ACTA) (Figure S4B), a TGFβ receptor ligand that drives skeletal muscle atrophy in cachexia.[23]

Next, we investigated whether the effect of FK506 on myotubes was due to its well-known activity on calcineurin activation. To this aim, we used cyclosporine A, another immunosuppressant drug that inhibits calcineurin in an FKBP12-independent manner.[48] C2C12 myotubes treated with cyclosporine A (CyA) undergo atrophy (Figure S4C), suggesting that the effect of FK506 on myotubes was independent of calcineurin inhibition.

In line with the increased myotube size in FK506-treated C2C12, FK506 alone strongly induced phosphorylation of Smad1/5 (Figure 3D) and synergized with murine BMP6 (Figures 3E and S4D). By performing BMP-responsive element luciferase reporter assay (BRE-Luc) in C2C12, we confirmed that, in cells treated with FK506, the luciferase signal was significantly higher than in controls (Figure 3F). This transcriptional upregulation was synergic with concomitant administration of BMP6, in line with increased pSmad1/5 (Figure 3E). These results demonstrated that the increased levels of pSmad1/5 were functionally active to target gene expression.

As FKBP12 blocks uncontrolled activation of the BMP pathway by binding BMPR-I,[49,50] we investigated which receptor accounted for BMP activation followed by FK506 treatment. We knocked- down with siRNA in C2C12 myotubes either ALK2

Figure 3. FK506 bypasses BMP pathway inhibition by activating pSmad1/5/8 through FKBP12 binding

(A) Representation of the proposed approach to bypass the "BMP resistance." FK506 binds FKBP12 displacing it from the BMPR-I and restoring pSmad1/5/8 signaling.

(B and C) Diameters quantification of C2C12 myotubes treated for 24 h with FK506 (1 μg/mL) and/or 10% C26 CM (B) (n = 5) or treated with recombinant Fc-ERFE (1 μg/mL) and FK506 in Fc-CTR (1 μg/mL) (C) (n = 4).

(D) Immunoblot of C2C12 myotubes serum starved for 2 h and treated with FK506 (1 μg/mL) for 1 h (n = 4).

(E) Dose-response and immunoblot of pSmad1/5/8 in C2C12 myotubes serum starved for 2 h and treated with increasing doses of murine BMP6 (from 0.01 to 3 ng/mL) with or without FK506 (1 μg/mL) for 1 h (n = 4).

(F) Luminescence signal detected in C2C12 transfected with pGL3-BMP responsive element (BRE)-Luciferase and TK-Renilla for 24 h, switched to low-serum DMEM 2% fetal bovine serum for 4 h and treated with FK506 (10 μg/mL) and BMP6 (30 ng/mL) for 5 h (n = 6).

(G) Immunoblot of pSmad1/5 and Smad1 of C2C12 myotubes knocked-down for ALK2 or ALK3 (siAlk2 or siAlk3) for 48 h and treated with FK506 (1 μg/mL) for 1 h upon 2 h of serum starvation (n = 2).

(H) mRNA levels of ALK2 and ALK3 in C2C12 myotubes knocked-down for ALK2 or ALK3 with siRNA (n = 2/3).

(I) Diameters of C2C12 myotubes treated with DMH1 (500 nM) in combination with FK506 (1 μg/mL) and/or 10% C26 CM for 24 h in differentiation medium (n = 3/4).

(J and K) *In vitro* SuNSET assay and relative quantifications performed with C2C12 myotubes treated with FK506 (1 μg/mL), 10% C26 CM (J) (n = 4) or Fc-CTR (1 μg/mL), Fc-ERFE (1 μg/mL), and inactive globular gERFE (1 μg/mL).

(K) (n = 3) For 24 h in differentiation medium. For the last 4 h of treatment, puromycin (1 μM) was added to the cells.

(L) Myotubes diameters imaged at 24 h post FK506 (1 μg/mL) and/or Torin1 (100 μM) treatment (n = 3).

(M and N) mRNA levels of Atrogin1 (M) (n = 3/5) and MuRF1 (N) (n = 3) in C2C12 myotubes treated for 48 h with FK506 (1 μg/mL) and/or 20% C26 CM. See also Figure S4. Data information: All graphs' statistical significance was tested with one-way ANOVA followed by Sidak's multiple comparison test.

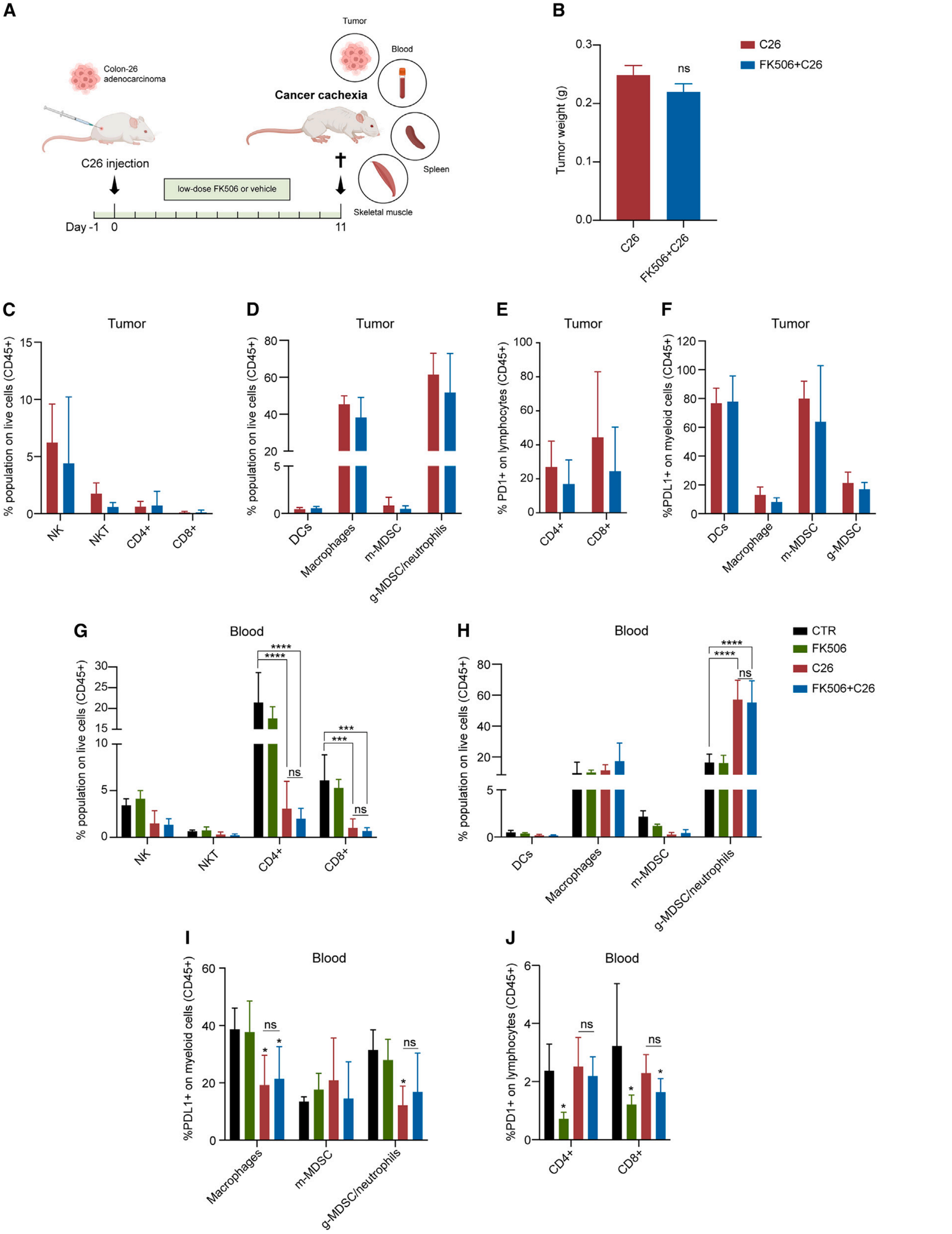

(legend on next page)

CellPress
OPEN ACCESS

or ALK3, two BMPR-I family members expressed in skeletal muscle, and only ALK2 downregulation reduced pSmad1/5 triggered by FK506 (Figures 3G and 3H). The relevance of ALK2 is furthermore proven when C2C12 myotubes were treated with dorsomorphin homolog 1 (DMH1), a BMPR-I inhibitor that primarily inhibits ALK2.[51] The inhibition of pSmad1/5 (Figure S4E) by DMH1 supplementation was sufficient to hinder the rescue of C26 CM-induced atrophy by FK506 (Figure 3I).

FK506 could activate the BMP-Smad axis by binding and displacing the intracellular inhibitor FKBP12 from BMPR-I family members.[33,52] To assess whether FKBP12 could interact with ALK2 or ALK3, we co-transfected HuH7 and 293T cells with MYC-tagged ALK2 or MYC-tagged ALK3 constructs with FLAG-tagged FKBP12 (Figures S4F and S4G).

In HuH7 cells, the interaction of FKBP12-FLAG is limited to ALK2 (Figure S3F)[33]; however, FKBP12-FLAG overexpression in 293T cells shows a minimal binding of FKBP12 to ALK3 (Figure S4G). In both cases, FK506 displaced FKBP12 from any BMPR-I analyzed (Figures S4F and S4G).

As the activation of the BMP-Smad1/5/8 axis has been associated to increased protein synthesis,[12,53] we performed a SuNSET assay to measure protein synthesis in our *in vitro* model of myotube atrophy. As expected, C2C12 treated C26 CM (Figures 3J and S4I), recombinant ERFE (Figures 3K and S4J), or ACTA (Figures S4H and S4K) and co-treated with FK506 showed significant higher levels of newly synthetized proteins (Figures 3J, 3K, and S4H–S4K), in line with C2C12 myotubes diameters (Figures 3B, 3C, and S4B).

Moreover, mammalian target of rapamycin (mTOR) inhibition by Torin 1 abolished the hypertrophy induced by FK506 (Figure 3L), demonstrating that the increase in myotube size is due to increased protein synthesis and it is mediated by mTOR. In line with these findings, we found that the atrophic markers Atrogin1 and MuRF1 were decreased in C26 CM-treated myotubes supplemented with FK506 (Figures 3M and 3N).

Overall, these results demonstrated that, *in vitro*, FK506 rescues myotube atrophy by inducing FKBP12 displacement from ALK2, increasing Smad1/5/8 signaling, ultimately leading to increased protein synthesis and decreased atrogenes levels.

FK506 effect on the immune system *in vivo*

To address the effect of FK506 in an *in vivo* cancer cachexia model, BALB/c mice were injected with C26 adenocarcinoma cells, and treated daily for 11 days (Figure 4A) with an oral low dose of FK506 (0.02 mg/kg) known to be non-immunosuppressive.[47] First, we verified whether the chosen dose of FK506 was altering the immune landscape and consequently the tumor size: after 11 days of treatment, tumor (Figure 4B) and spleen weights (Figure S5A) were unchanged, indicating that our treatment with FK506 does not exert an antitumoral or an anti-inflammatory effect.[54]

In addition, we performed an analysis of immune cell populations in tumors, blood, and spleens of treated and control mice. No significant differences were found, as the amount of the main lymphoid and myeloid populations, such as natural killer, natural killer T, $CD4^+$ and $CD8^+$ T cells, dendritic cells, macrophages, monocytic myeloid-derived suppressor cells (m-MDSC) and granulocyte-like MDSC (g-MDSC), was similar in the tumors from control and FK506-treated mice (Figures 4C and 4D). Moreover, FK506 treatment did not alter the expression of programmed cell death 1 (PD-1) on T lymphocytes (Figure 4E) or that of programmed cell death ligand 1 (PD-L1) on myeloid cells (Figure 4F). As expected, tumor-bearing mice showed significant decrease in blood and splenic $CD4^+$ and $CD8^+$ T cells (Figures 4G and S5B), increased splenic macrophages (Figure S5C), as well as increased g-MDSC/neutrophils populations in the blood (Figure 4H), independent of treatment with FK506. Furthermore, C26 tumor-bearing mice displayed a significant decrease of PD-L1-positive macrophages and g-MDSC in the blood (Figure 4I), and an increase of PD-1-expressing $CD4^+$ and $CD8^+$ T cells and of PD-$L1^+$ m-MDSC in the spleen (Figures S5D and S5E), again regardless of FK506 administration.

Despite FK506 decreased PD-1-expressing $CD4^+$ and $CD8^+$ T cells in the blood of control mice, these alterations were not observed in C26 tumor-bearing mice (Figure 4J), suggesting that FK506 does not affect tumor immune escape. Nonetheless, we sought to further exclude whether chronic low-dose FK506 treatment could disturb adaptive immune responses in tumor-free mice. We thus vaccinated with a non-self antigen (RhuT vaccine)[55] to induce a strong immune response. All mice were treated for 5 weeks with daily oral gavage of low-dose FK506 or vehicle until the end of the experiment (Figure S5F). To test if FK506 affected specific lymphocyte functions and activities, we evaluated whether long-term FK506 treatment modified antibody production or immune cell cytotoxic capacity. After 5 weeks of treatment, antibody titers against the antigen (Figure S5G), as well as activation of $CD8^+$ T cells (Figure S5H) were not different in either FK506-treated or -untreated vaccinated mice. In agreement, this long chronic treatment with FK506 did not significantly alter the amount of blood lymphoid populations and the expression of the activation marker PD-1 (Figure S5I and S5J).

These findings indicated that prolonged low-dose FK506 treatment does not significantly alter the amount and function of immune cells in a tumor-free mouse model, corroborating that low-dose FK506 does not impact adaptive immunity.

Figure 4. FK506 effect on the immune system *in vivo*

(A) Experimental design of the *in vivo* C26 cancer cachexia model orally treated with low-dose FK506 (0.02 mg/kg) or vehicle for 11 days. When euthanized, tumors, blood, spleens, and skeletal muscles were analyzed.

(B) Tumor weight of FK506-treated and vehicle-treated C26 tumor-bearing mice 11 days post-C26 injection (n = 16).

(C and D) Percentages of lymphoid (C) and myeloid (D) populations on live cells ($CD45^+$) in the tumors of FK506 or vehicle-treated C26 tumor-bearing mice (n = 6).

(E and F) Percentage of PD-1-expressing lymphocytes (E) and PD-L1+ myeloid cells (F) in C26 tumors of FK506 and vehicle-treated mice (n = 6).

(G and H) Amount of blood lymphoid (G) and myeloid (H) populations in FK506 and vehicle-treated tumor and non-tumor-bearing mice at day 11 post-C26 injection (n = 4/6).

(I and J) Percentage of PD-L1+ myeloid cells (I) and PD-1+ lymphocytes (J) in the blood of the experimental groups 11 days post-C26 inoculation (n = 4/6) See also Figure S5. Data information: Statistical significance was tested with unpaired two-tailed Student's *t*-test in (B) and with two-way ANOVA followed by Sidak's multiple comparison test in (C)–(J); ns, not significant.

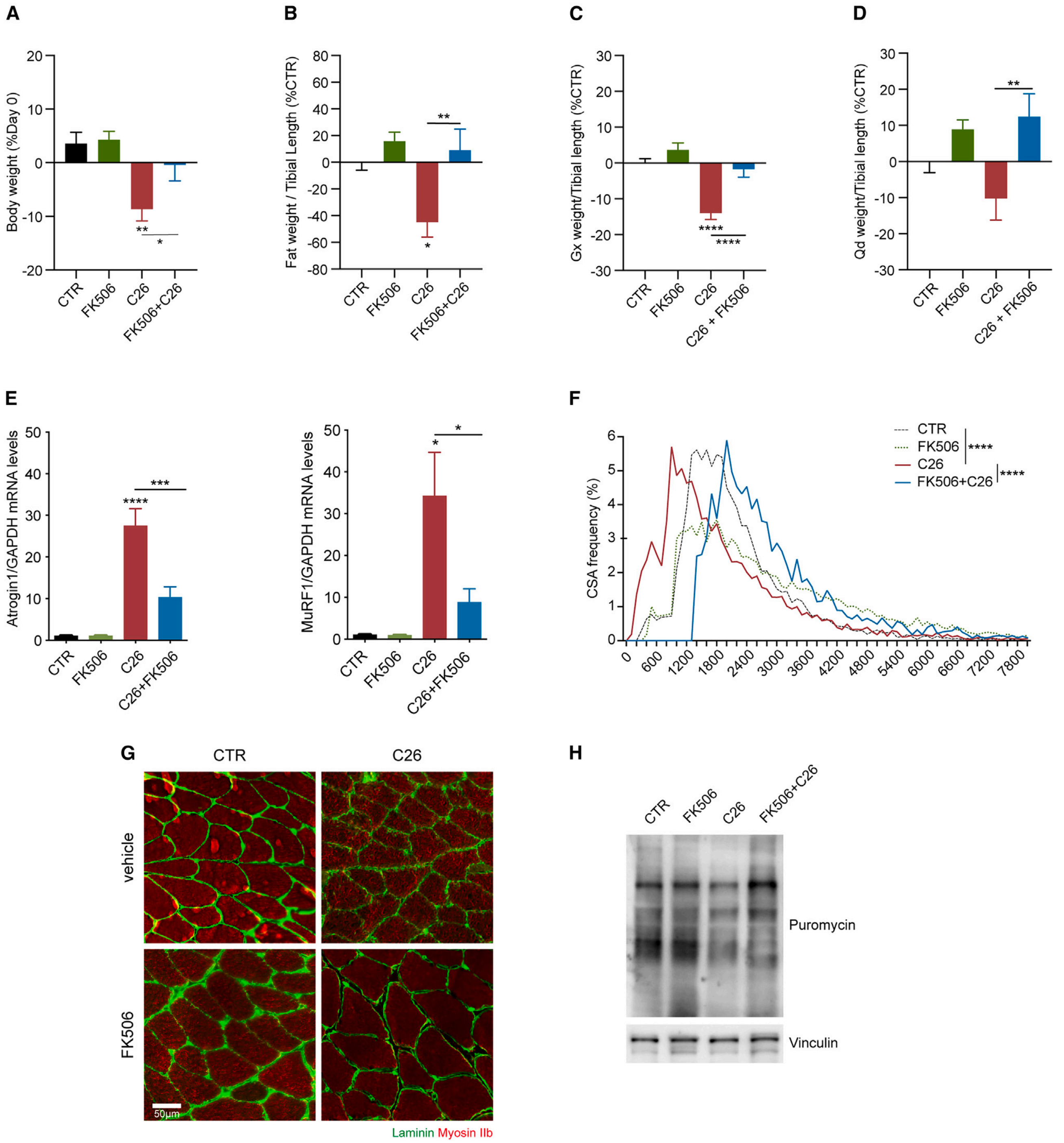

Figure 5. FK506 protects from cachexia in C26 tumor-bearing mice

(A) Body weight change as percentage of initial body weight in the experimental groups at day 11 (n = 10/12).

(B) Fat weight normalized for the tibial length as percentage of non-tumor-bearing vehicle-treated mice at day 11 (n = 6/7).

(C and D) Gastrocnemii (C) and quadriceps (D) weights normalized for tibial length as percentage of the control group 11 days post-C26 inoculation (n = 15/16 for gastrocnemii and n = 11/12 for quadriceps).

(E) Expression levels of the atrogenes Atrogin1 and MuRF1 in the gastrocnemii of the experimental groups at day 11 (n = 4/6).

(legend continued on next page)

FK506 protects from cachexia in C26 tumor-bearing mice

Since low-dose FK506 was found not to be immunosuppressive, we analyzed the effects of FK506 in cachexia. FK506-treated C26 tumor-bearing mice appeared healthier and less distressed than the untreated group (Video S1), as seen by the preservation of body weight (Figure 5A) and fat tissue weight (Figure 5B) at the endpoint of the experiment. Furthermore, the acute and severe skeletal muscle atrophy induced by C26 tumors was reverted upon FK506 treatment, as indicated by the increased muscle weights (Figures 5C and 5D) and by the lower levels of atrogenes expression (Figures 5E, S6A, and S6B). In agreement with increased muscle weights, fibers CSA in the treated group were significantly higher than in controls (Figures 5F and 5G). Since the tumor weight in FK506-treated and vehicle-treated mice was unchanged (Figure 4B), protection against muscle wasting was not due to decreased tumor growth.

To assess whether the beneficial effect of FK506 was due also to increased protein synthesis, we performed the *in vivo* SuNSET assay. As shown in Figure 5H, the reduced amount of newly synthetized proteins in atrophic muscles from tumor-bearing mice was fully rescued in FK506-treated samples (Figures 5H and S6C), in further support to rescued CSA fiber size (Figure 5F). Overall, these findings demonstrate that a low dose of FK506 prevents skeletal muscle wasting and cancer cachexia *in vivo* (Figures 5 and S6).

FK506 prevents the degeneration of NMJs morphology and preserves muscle strength

An important component of cachexia-induced skeletal muscle atrophy is the degeneration of the neuromuscular compartment.[14] Indeed, together with skeletal muscle atrophy, the decrease in muscle force in C26 tumor-bearing mice is prevented by FK506 treatment (Figure 6A).

In agreement with the decreased muscle strength, cachectic mice showed smaller NMJs with a collapsed morphology (Figures 6B and 6C) and downregulation of the adult nicotinic acetylcholine receptor subunit *Chrne* mRNA (Figure 6D). In contrast, FK506 treatment rescued NMJs volume, morphology, and *Chrne* expression levels (Figures 6B–6D).

Considering the pre-synaptic compartment of the NMJs, we evaluated the denervation status in our C26 model, where we only found a trend in denervated NMJs (Figure S6D). However, molecular alterations (i.e., *Chrne* downregulation), and the reduction in NMJs volume, suggest an ongoing remodeling, but partially occurring, degenerative process, largely attributed to the rapid kinetics of the C26 cell suspension cachexia model.

DISCUSSION

Patients with cancer and chronic inflammatory diseases commonly develop cachexia, a multiorgan condition characterized by body weight loss, fat, and skeletal muscle wasting.[1] Although cachexia is related to anticancer treatment intolerance, worsening of prognosis, and death of advanced cancer patients,[3,4] it remains incurable and poorly characterized. From a physiological standpoint, one of the main signaling pathway having a role in controlling skeletal muscle mass is the TGFβ superfamily, where activin/TGFβ-Smad2/3 signaling induces atrophy[21–25] and it is counteracted by the opposite BMP-Smad1/5/8 axis. The former indeed drives atrogenes expression and protein breakdown, while the latter induces protein synthesis.[12,13]

Our results demonstrated that, in cancer cachexia, a state of systemic inflammation contributes to the upregulation of the BMP scavenger ERFE through STAT3 activation. This extracellular inhibition is combined with the activity of the immunophilin FKBP12, which acts as an intracellular brake for BMP signaling activation. These two layers of inhibition contribute to decreased activation of pSmad1/5/8 in atrophic muscles and cachexia.

FK506 bypasses this state of BMP resistance by displacing FKBP12 from BMPR-I, restoring pSmad1/5/8 signaling *in vitro*, increasing protein synthesis, and preventing the degeneration of skeletal muscle functionality *in vivo* (Figure 6E).

We thus identified two inhibitors of the BMP signaling as mediators of muscle atrophy, previously characterized in the field of iron metabolism, i.e., the extracellular BMP ligand chelator ERFE and the intracellular inhibitor of the BMPR FKBP12. ERFE is a hormone produced by erythroid cells known to reduce Smad1/5/8 signaling in the liver by sequestering BMPs from their receptors.[28,30,31] Notably, although ERFE is also expressed in the skeletal muscle (and named myonectin[32]), its biological function and mechanism of regulation in the muscle remains to be clarified. In contrast, FKBP12 is a peptidyl-prolyl-cis-trans cytosolic isomerase that belongs to the immunophilin superfamily, known to form various heterocomplexes, as its binding to the glycine-serine–rich domain of BMPR-I avoids their uncontrolled activation.[49,50]

Our results demonstrated that, in cachectic muscles, combined with the already known hyperactivation of the Smad2/3 axis,[21–25] a BMP resistance characterized by decreased BMP signaling is observed despite similar levels of BMP ligands. We found indeed that the BMP inhibitor ERFE is more expressed in models of cancer cachexia and in muscles of pre-cachectic and cachectic cancer patients. This indicates that some muscular alterations can precede the onset of cachexia, potentially contributing to its development. Of note, it has been even reported that conditions such as metabolic rewiring and muscle wasting predate the diagnosis of cancer.[9,56,57]

We found that phosphorylated STAT3 (Tyr705) is recruited to putative STAT3 binding sites on the promoter of *Erfe* in cachectic muscles, and that its inhibition or knockdown is sufficient to prevent ERFE upregulation. STAT3 activation in the skeletal muscle is a key signaling pathway that drives wasting and cachexia,[38] also due to the high levels of IL-6 in cachectic patients and mice.[40,58–60]

(F and G) Frequencies distribution of the CSA of the fibers in the gastrocnemii of vehicle and FK506-treated control and C26 tumor-bearing mice and representative immunofluorescence pictures of gastrocnemii stained with Laminin (green) and myosin IIb (red) (G) (n = 4/5).
(H) *In vivo* SuNSET assay and immunoblot for puromycin performed with the gastrocnemii of the different groups (n = 3/4). See also Figure S6. Data information: Statistical significance was tested with one-way ANOVA followed by Sidak's multiple comparison test in (A)–(E) and with two-way ANOVA followed by Sidak's multiple comparison test in (F).

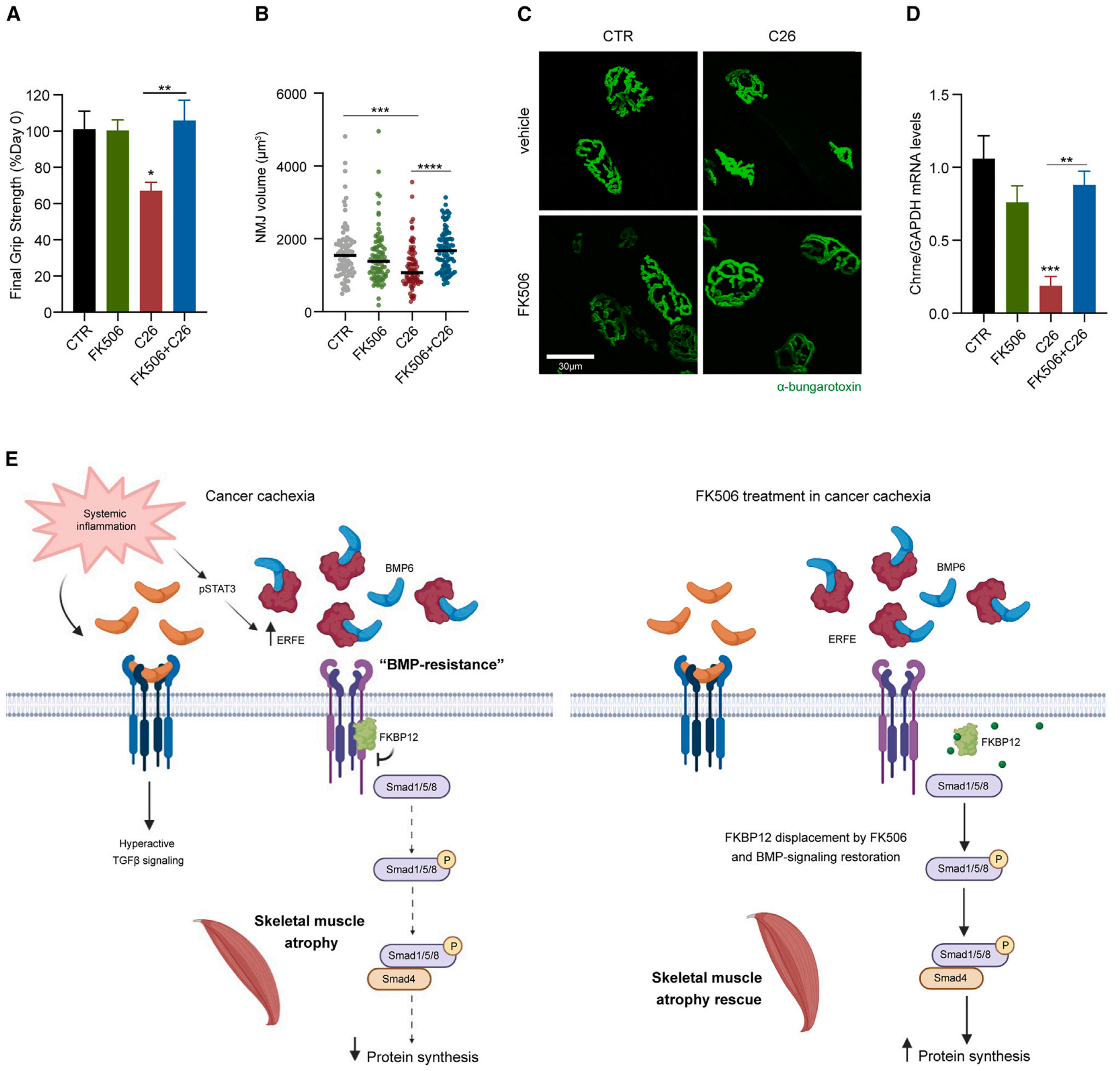

Figure 6. FK506 prevents the degeneration of NMJs morphology and preserves muscle strength

(A) Final grip strength as percentage of initial grip strength at day 0 in FK506 or vehicle-treated C26 tumor-bearing and control mice at day 11 post-C26 injection (n = 5/7).

(B and C) Volume of the NMJs quantified in the EDL muscle and stained with α-bungarotoxin-Alexa 488 (B) and relative representative picture of the experimental groups (C) (n = 3, 28–30 NMJs quantified per mouse).

(D) Expression levels of *Chrne* gene in the gastrocnemii of FK506 or vehicle treated C26 and control mice at day 11 post-C26 injection (n = 3/4).

(E) Graphical representation of the molecular mechanism. In cancer cachexia, systemic inflammation induces catabolism by hyperactivating TGFβ-Smad2/3 axis and by increasing the expression of BMP signaling inhibitors. Activated pSTAT3 induces the upregulation of the BMP scavenger ERFE, which in combination with the intracellular inhibitor FKBP12, contributes to the downregulation of the BMP-pSmad1/5/8 axis and therefore reducing protein synthesis in atrophic muscles. Our therapeutic approach proposes the use of low-dose FK506, that by binding the immunophilin FKBP12 induces its displacement from the BMPR-I, rescuing pSmad1/5/8 signaling and protecting from muscle atrophy in cancer cachexia. See also Figure S6. Data information: Statistical significance was tested with one-way ANOVA followed by Sidak's multiple comparison test.

We found that C26 CM, as well as IL-6, induced pSTAT3. The finding that stimulation with IL-6 was weaker than with CM could indicate that multiple cytokines can contribute to STAT3 activation in the C26 model. Indeed, while the tumor is undoubtedly a source of inflammatory cytokines and factors known to activate STAT3,[61] other compartments may also contribute to the chronic and systemic inflammation in cachexia. For instance, tumor-related dysbiosis[62] and altered gut permeability and endotoxemia[63,64] are known to promote STAT3 activation and cancer progression.[65]

The functional relevance of the inhibitors ERFE and FKBP12 was subsequently characterized in *in vitro* and *in vivo* models of cachexia. ERFE supplementation significantly reduced myotube diameter, while *Fkbp12* knockdown protected from atrophy in C2C12 myotubes. Similarly, muscle-specific AAV-mediated delivery of shRNA against *Erfe* or *Fkbp12* was sufficient to decrease muscle atrophy induced by C26 tumor. These results are coherent with a previous study showing that Noggin, another BMP inhibitor, is upregulated and induces atrophy and denervation in cachexia.[14] In contrast, extracellular activators of the catabolic signaling exerted by pSmad2/3, as myostatin and activin A have been found to be highly expressed in cachectic patients and to induce muscle wasting in various murine models of cachexia.[21,23,25,66,67] However, the targeting of myostatin failed to be effective in cancer patients, leading to the suspension of the clinical study (LY2495655)[68] and pointing to the necessity of alternative targets.

A cause for this failure might be due to the redundancy of extracellular and intracellular TGFβ superfamily members. Therefore, targeting a single interactor might not be sufficient to generate a relevant biological effect. To bypass the redundancy and the extracellular ligands competition, we targeted the intracellular protein FKBP12 to restore BMPR signaling.

In the absence of TGFβ superfamily ligands, FKBP12 binds BMPR-I,[36,69] decreasing their signaling,[50,70] while high levels of BMP6 induce FKBP12 displacement from ALK2 receptor in hepatoma cells.[33]

We found that FK506 fully rescues myotube atrophy, by activating pSmad1/5/8 axis and inducing protein synthesis *in vitro*. Interestingly, we observed the same effects in C26 tumor-bearing mice, where FK506-treatment reversed body weight loss and muscle atrophy by increasing fiber size and protein synthesis, and by preventing NMJs alterations.

Once upregulated, FKBP12 can bind several type I TGFβ superfamily receptors.[49,52] In 293T cells, the supraphysiological expression of FKBP12 promotes moderate interaction with ALK3. However, in our study we found that FKBP12 preferentially binds ALK2, as in previous studies on hepatic Smad signaling regulation.[33,34] Hence, ALK2 knockdown in C2C12 myotubes reduces FK506-induced Smad1/5 phosphorylation, and its inhibition blunts the rescue of atrophy observed with FK506. Beyond its promiscuity to BMPR-I, FKBP12 has also been demonstrated to bind other proteins forming ternary structures as the rapamycin-FKBP12-mTOR complex. When FKBP12 is bound to rapamycin, the complex inhibits mTOR, with an immunosuppressive effect by blocking protein synthesis.[71] In our context, we exclude any off-target effects on mTOR inhibition by FK506, as we observed increased protein synthesis upon FK506 treatment *in vitro* and *in vivo*, while mTOR inhibition by Torin1 blunts the hypertrophy induced by FK506 in C2C12 myotubes.

Given the immunosuppressive action of FK506, we tested the effect of low doses to maintain immune responses.[47] In agreement, no alterations in the immunological landscape were present in mice treated with either long- or short-term FK506 treatment. Further indications that FK506 effect is independent from immunosuppression are represented by the curb of atrophy observed by direct FKBP12 knocked- down in muscles. Moreover, cyclosporine A, an inhibitor of calcineurin independent from FKBP12, induces atrophy in C2C12 myotubes.

An emerging feature of muscle wasting in cancer is the progressive loss of NMJs integrity due to reduced Smad1/5/8 signaling.[14] This pathway has indeed been shown to be involved in NMJs fitness and functionality,[14,26,27] suggesting that FK506 might contribute to maintain the cross-talk between the skeletal muscle and the NMJs through the BMP-Smad1/5/8 signaling. In agreement, we found that FK506 preserved the volume and morphology of the NMJs in C26 tumor-bearing mice and reverted the downregulation of the adult epsilon subunit of the nicotinic acetylcholine receptor, a known marker of functional adult NMJs.[72]

The use of a potentially immunosuppressive drug in cancer patients might be counterintuitive and would require fine dose optimization in humans to avoid undesired immunosuppression. Despite these limitations, the off-label use of an approved drug significantly decreases the costs of development of a new molecule, as its safety and toxicity are already known. Moreover, further studies in a slower paced cachexia will be important to move closer to human physiology. As previously described, cachexia affects patients with chronic inflammatory diseases like cancer, chronic obstructive pulmonary disease (COPD), AIDS, and sepsis, and it still represents an unmet medical need.[2,73] Our results on improved muscle functionality and protection from NMJs alterations can expand this therapeutic approach to neuromuscular diseases independent of cachexia.

Limitations of the study

We acknowledge some limitations to our study. The study has been performed mostly with a specific murine cancer model, i.e., the C26, which is an injectable tumor cells suspension characterized by a rapid development of cachexia. Hence, to understand the global impact of FK506 treatment on cancer cachexia, it would be important the use of models with slower kinetic, such as GEMMs, or in combination with clinically relevant treatments such as chemotherapy. It would also be interesting to determine whether our characterization is specific to the C26 model, or the features are also shared in other tumor models.

Considering the translatability in the clinic, the use of an immunosuppressive drug as FK506 could be critical in cancer patients. Therefore, the identification and optimization of the effective dose of FK506 to trigger Smad1/5/8 pathway without providing immunosuppression in humans would be required.

Finally, even though we found that the hormone ERFE is upregulated in human pre-cachectic and cachectic muscle biopsies, longitudinal studies in cancer patients are required to determine

causality and the identification of ERFE as potential prognostic marker.

STAR★METHODS

Detailed methods are provided in the online version of this paper and include the following:

- KEY RESOURCES TABLE
- RESOURCE AVAILABILITY
  - Lead contact
  - Materials availability
  - Data and code availability
- EXPERIMENTAL MODEL AND SUBJECT DETAILS
  - Human skeletal muscles biopsies
  - Animals
  - Cell lines
- METHOD DETAILS
  - Cell culture
  - Myotube diameter measurement
  - siRNA-induced knockdown
  - Luciferase assay
  - Western blotting
  - *In vitro* co-immunoprecipitation (Co-IP)
  - Animal experimentation
  - Eythroferrone enzyme-linked immunosorbent assay (ELISA)
  - Protein synthesis measurement with SuNSET assay
  - C2C12 myotubes and skeletal muscles chromatin immunoprecipitation (ChIP-qPCR)
  - Histology
  - Neuromuscular junction analysis
  - RNA isolation and quantitative PCR (qPCR)
  - Adeno-associated virus (AAV) shRNA cloning
  - FACS
  - Vaccine preparation and mouse immunization
  - Antibody response
  - IFN-γ enzyme-linked immunospot (ELISpot) assay
- QUANTIFICATION AND STATISTICAL ANALYSIS

SUPPLEMENTAL INFORMATION

Supplemental information can be found online at https://doi.org/10.1016/j.xcrm.2023.101306.

ACKNOWLEDGMENTS

The research leading to these results has received funding from AIRC under MFAG 2018 - ID. 21564 project – P.I. Porporato Paolo Ettore; PoC Instrument 2021; PoC ToInProVE2; Consorzio Interuniversitario per le Biotecnologie (CIB); and AIRC under MFAG 2022 ID. 25908 project – P.I. Alessio Menga. European Research Council (ERC) under the European Union’s Horizon 2020 research and innovation program (grant agreement no. 715491) to P.I. Léon Kautz and Project Age-it, PE0000015, PNRR MUR – M4C2 1.3 funded by Next-Generation EU to P.I. Andrea Graziani. V.R. fellowship was funded by AIRC.

The authors are thankful to Alessandro Bertero, Matteo Biolatti, and Fabio Penna for helpful discussion and to Alessandra Ghigo, Lidia Avalle, Natalia Cortez-Penso, and Chiara Ambrogio for technical support.

AUTHOR CONTRIBUTIONS

Conceptualization: E.M. and P.E.P.; formal analysis: E.M., R.S, A.M., and P.E.P.; investigation: E.M., E.W., R.S., L.C., V.R., I.Z., M.M., and E.A.; resources: A.P., L.S., A.G., L.K., S.O., E.H., M.H., A.F., C.S., and M.S.; data curation: E.M.; writing – original draft: E.M. and P.E.P.; supervision: P.E.P.

DECLARATION OF INTERESTS

E.M., V.R., L.S., and P.E.P. declare to be inventors of the patent PCTIB2022050175 (WO2022/149113).

Received: February 22, 2023
Revised: September 29, 2023
Accepted: November 7, 2023
Published: December 4, 2023

REFERENCES

1. Fearon, K., Strasser, F., Anker, S.D., Bosaeus, I., Bruera, E., Fainsinger, R.L., Jatoi, A., Loprinzi, C., MacDonald, N., Mantovani, G., et al. (2011). Definition and classification of cancer cachexia: an international consensus. Lancet Oncol. *12*, 489–495.
2. von Haehling, S., and Anker, S.D. (2010). Cachexia as a major underestimated and unmet medical need: facts and numbers. J Cachexia Sarcopenia Muscle *1*, 1–5.
3. Fearon, K.C. (2011). Cancer cachexia and fat-muscle physiology. N. Engl. J. Med. *365*, 565–567.
4. Rausch, V., Sala, V., Penna, F., Porporato, P.E., and Ghigo, A. (2021). Understanding the common mechanisms of heart and skeletal muscle wasting in cancer cachexia. Oncogenesis *10*, 1.
5. Zhou, X., Wang, J.L., Lu, J., Song, Y., Kwak, K.S., Jiao, Q., Rosenfeld, R., Chen, Q., Boone, T., Simonet, W.S., et al. (2010). Reversal of cancer cachexia and muscle wasting by ActRIIB antagonism leads to prolonged survival. Cell *142*, 531–543.
6. Ballaro, R., Beltra, M., De Lucia, S., Pin, F., Ranjbar, K., Hulmi, J.J., Costelli, P., and Penna, F. (2019). Moderate exercise in mice improves cancer plus chemotherapy-induced muscle wasting and mitochondrial alterations. FASEB J *33*, 5482–5494.
7. Wyart, E., Hsu, M.Y., Sartori, R., Mina, E., Rausch, V., Pierobon, E.S., Mezzanotte, M., Pezzini, C., Bindels, L.B., Lauria, A., et al. (2022). Iron supplementation is sufficient to rescue skeletal muscle mass and function in cancer cachexia. EMBO Rep. *23*, e53746.
8. Wang, G., Biswas, A.K., Ma, W., Kandpal, M., Coker, C., Grandgenett, P.M., Hollingsworth, M.A., Jain, R., Tanji, K., Lomicronpez-Pintado, S., et al. (2018). Metastatic cancers promote cachexia through ZIP14 upregulation in skeletal muscle. Nat Med *24*, 770–781.
9. Brown, J.L., Rosa-Caldwell, M.E., Lee, D.E., Blackwell, T.A., Brown, L.A., Perry, R.A., Haynie, W.S., Hardee, J.P., Carson, J.A., Wiggs, M.P., et al. (2017). Mitochondrial degeneration precedes the development of muscle atrophy in progression of cancer cachexia in tumour-bearing mice. J Cachexia Sarcopenia Muscle *8*, 926–938.
10. Wyart, E., Reano, S., Hsu, M.Y., Longo, D.L., Li, M., Hirsch, E., Filigheddu, N., Ghigo, A., Riganti, C., and Porporato, P.E. (2018). Metabolic Alterations in a Slow-Paced Model of Pancreatic Cancer-Induced Wasting. Oxid. Med. Cell. Longev. *2018*, 6419805.
11. Sartori, R., Milan, G., Patron, M., Mammucari, C., Blaauw, B., Abraham, R., and Sandri, M. (2009). Smad2 and 3 transcription factors control muscle mass in adulthood. Am J Physiol Cell Physiol *296*, C1248–C1257.
12. Winbanks, C.E., Chen, J.L., Qian, H., Liu, Y., Bernardo, B.C., Beyer, C., Watt, K.I., Thomson, R.E., Connor, T., Turner, B.J., et al. (2013). The bone morphogenetic protein axis is a positive regulator of skeletal muscle mass. J. Cell Biol. *203*, 345–357.

13. Sartori, R., Schirwis, E., Blaauw, B., Bortolanza, S., Zhao, J., Enzo, E., Stantzou, A., Mouisel, E., Toniolo, L., Ferry, A., et al. (2013). BMP signaling controls muscle mass. Nat. Genet. *45*, 1309–1318.
14. Sartori, R., Hagg, A., Zampieri, S., Armani, A., Winbanks, C.E., Viana, L.R., Haidar, M., Watt, K.I., Qian, H., Pezzini, C., et al. (2021). Perturbed BMP signaling and denervation promote muscle wasting in cancer cachexia. Sci. Transl. Med. *13*.
15. Winbanks, C.E., Murphy, K.T., Bernardo, B.C., Qian, H., Liu, Y., Sepulveda, P.V., Beyer, C., Hagg, A., Thomson, R.E., Chen, J.L., et al. (2016). Smad7 gene delivery prevents muscle wasting associated with cancer cachexia in mice. Sci. Transl. Med. *8*, 348ra398.
16. Sandri, M., Sandri, C., Gilbert, A., Skurk, C., Calabria, E., Picard, A., Walsh, K., Schiaffino, S., Lecker, S.H., and Goldberg, A.L. (2004). Foxo transcription factors induce the atrophy-related ubiquitin ligase atrogin-1 and cause skeletal muscle atrophy. Cell *117*, 399–412.
17. Schiaffino, S., Dyar, K.A., Ciciliot, S., Blaauw, B., and Sandri, M. (2013). Mechanisms regulating skeletal muscle growth and atrophy. FEBS J. *280*, 4294–4314.
18. Lecker, S.H., Jagoe, R.T., Gilbert, A., Gomes, M., Baracos, V., Bailey, J., Price, S.R., Mitch, W.E., and Goldberg, A.L. (2004). Multiple types of skeletal muscle atrophy involve a common program of changes in gene expression. FASEB J *18*, 39–51.
19. Huang, J., and Zhu, X. (2016). The molecular mechanisms of calpains action on skeletal muscle atrophy. Physiol. Res. *65*, 547–560.
20. Mammucari, C., Milan, G., Romanello, V., Masiero, E., Rudolf, R., Del Piccolo, P., Burden, S.J., Di Lisi, R., Sandri, C., Zhao, J., et al. (2007). FoxO3 controls autophagy in skeletal muscle in vivo. Cell Metab *6*, 458–471.
21. Loumaye, A., de Barsy, M., Nachit, M., Lause, P., Frateur, L., van Maanen, A., Trefois, P., Gruson, D., and Thissen, J.P. (2015). Role of Activin A and myostatin in human cancer cachexia. J. Clin. Endocrinol. Metab. *100*, 2030–2038.
22. Loumaye, A., de Barsy, M., Nachit, M., Lause, P., van Maanen, A., Trefois, P., Gruson, D., and Thissen, J.P. (2017). Circulating Activin A predicts survival in cancer patients. J Cachexia Sarcopenia Muscle *8*, 768–777.
23. Chen, J.L., Walton, K.L., Winbanks, C.E., Murphy, K.T., Thomson, R.E., Makanji, Y., Qian, H., Lynch, G.S., Harrison, C.A., and Gregorevic, P. (2014). Elevated expression of activins promotes muscle wasting and cachexia. FASEB J *28*, 1711–1723.
24. Lee, S.J., and McPherron, A.C. (1999). Myostatin and the control of skeletal muscle mass. Curr. Opin. Genet. Dev. *9*, 604–607.
25. Zimmers, T.A., Davies, M.V., Koniaris, L.G., Haynes, P., Esquela, A.F., Tomkinson, K.N., McPherron, A.C., Wolfman, N.M., and Lee, S.J. (2002). Induction of cachexia in mice by systemically administered myostatin. Science *296*, 1486–1488.
26. Ball, R.W., Warren-Paquin, M., Tsurudome, K., Liao, E.H., Elazzouzi, F., Cavanagh, C., An, B.S., Wang, T.T., White, J.H., and Haghighi, A.P. (2010). Retrograde BMP signaling controls synaptic growth at the NMJ by regulating trio expression in motor neurons. Neuron *66*, 536–549.
27. Lee, S.H., Kim, Y.J., and Choi, S.Y. (2016). BMP signaling modulates the probability of neurotransmitter release and readily releasable pools in Drosophila neuromuscular junction synapses. Biochem. Biophys. Res. Commun. *479*, 440–446.
28. Kautz, L., Jung, G., Valore, E.V., Rivella, S., Nemeth, E., and Ganz, T. (2014). Identification of erythroferrone as an erythroid regulator of iron metabolism. Nat. Genet. *46*, 678–684.
29. Arezes, J., Foy, N., McHugh, K., Sawant, A., Quinkert, D., Terraube, V., Brinth, A., Tam, M., LaVallie, E.R., Taylor, S., et al. (2018). Erythroferrone inhibits the induction of hepcidin by BMP6. Blood *132*, 1473–1477.
30. Wang, C.Y., Xu, Y., Traeger, L., Dogan, D.Y., Xiao, X., Steinbicker, A.U., and Babitt, J.L. (2020). Erythroferrone lowers hepcidin by sequestering BMP2/6 heterodimer from binding to the BMP type I receptor ALK3. Blood *135*, 453–456.
31. Kautz, L., Jung, G., Du, X., Gabayan, V., Chapman, J., Nasoff, M., Nemeth, E., and Ganz, T. (2015). Erythroferrone contributes to hepcidin suppression and iron overload in a mouse model of beta-thalassemia. Blood *126*, 2031–2037.
32. Seldin, M.M., Peterson, J.M., Byerly, M.S., Wei, Z., and Wong, G.W. (2012). Myonectin (CTRP15), a novel myokine that links skeletal muscle to systemic lipid homeostasis. J. Biol. Chem. *287*, 11968–11980.
33. Colucci, S., Pagani, A., Pettinato, M., Artuso, I., Nai, A., Camaschella, C., and Silvestri, L. (2017). The immunophilin FKBP12 inhibits hepcidin expression by binding the BMP type I receptor ALK2 in hepatocytes. Blood *130*, 2111–2120.
34. Pettinato, M., Dulja, A., Colucci, S., Furiosi, V., Fette, F., Steinbicker, A.U., Muckenthaler, M.U., Nai, A., Pagani, A., and Silvestri, L. (2023). FKBP12 inhibits hepcidin expression by modulating BMP receptors interaction and ligand responsiveness in hepatocytes. Am. J. Hematol. *98*, 1223–1235.
35. Song, G.A., Kim, H.J., Woo, K.M., Baek, J.H., Kim, G.S., Choi, J.Y., and Ryoo, H.M. (2010). Molecular consequences of the ACVR1(R206H) mutation of fibrodysplasia ossificans progressiva. J. Biol. Chem. *285*, 22542–22553.
36. Wang, T., Li, B.Y., Danielson, P.D., Shah, P.C., Rockwell, S., Lechleider, R.J., Martin, J., Manganaro, T., and Donahoe, P.K. (1996). The immunophilin FKBP12 functions as a common inhibitor of the TGF beta family type I receptors. Cell *86*, 435–444.
37. Mathelier, A., Fornes, O., Arenillas, D.J., Chen, C.Y., Denay, G., Lee, J., Shi, W., Shyr, C., Tan, G., Worsley-Hunt, R., et al. (2016). JASPAR 2016: a major expansion and update of the open-access database of transcription factor binding profiles. Nucleic Acids Res. *44*, D110–D115.
38. Bonetto, A., Aydogdu, T., Kunzevitzky, N., Guttridge, D.C., Khuri, S., Koniaris, L.G., and Zimmers, T.A. (2011). STAT3 activation in skeletal muscle links muscle wasting and the acute phase response in cancer cachexia. PLoS One *6*, e22538.
39. Narsale, A.A., and Carson, J.A. (2014). Role of interleukin-6 in cachexia: therapeutic implications. Curr. Opin. Support. Palliat. Care *8*, 321–327.
40. Strassmann, G., Fong, M., Kenney, J.S., and Jacob, C.O. (1992). Evidence for the involvement of interleukin 6 in experimental cancer cachexia. J. Clin. Invest. *89*, 1681–1684.
41. Zimmers, T.A., Fishel, M.L., and Bonetto, A. (2016). STAT3 in the systemic inflammation of cancer cachexia. Semin. Cell Dev. Biol. *54*, 28–41.
42. Zhang, T., Zhang, Z., Dong, Q., Xiong, J., and Zhu, B. (2020). Histone H3K27 acetylation is dispensable for enhancer activity in mouse embryonic stem cells. Genome Biol. *21*, 45.
43. Schust, J., Sperl, B., Hollis, A., Mayer, T.U., and Berg, T. (2006). Stattic: a small-molecule inhibitor of STAT3 activation and dimerization. Chem Biol *13*, 1235–1242.
44. Arezes, J., Foy, N., McHugh, K., Quinkert, D., Benard, S., Sawant, A., Frost, J.N., Armitage, A.E., Pasricha, S.R., Lim, P.J., et al. (2020). Antibodies against the erythroferrone N-terminal domain prevent hepcidin suppression and ameliorate murine thalassemia. Blood *135*, 547–557.
45. Sato, Y., Fu, Y., Liu, H., Lee, M.Y., and Shaw, M.H. (2021). Tumor-immune profiling of CT-26 and Colon 26 syngeneic mouse models reveals mechanism of anti-PD-1 response. BMC Cancer *21*, 1222.
46. Hogan, P.G., Chen, L., Nardone, J., and Rao, A. (2003). Transcriptional regulation by calcium, calcineurin, and NFAT. Genes Dev. *17*, 2205–2232.
47. Tampe, B., Tampe, D., Nyamsuren, G., Klopper, F., Rapp, G., Kauffels, A., Lorf, T., Zeisberg, E.M., Muller, G.A., Kalluri, R., et al. (2018). Pharmacological induction of hypoxia-inducible transcription factor ARNT attenuates chronic kidney failure. J. Clin. Invest. *128*, 3053–3070.
48. Ho, S., Clipstone, N., Timmermann, L., Northrop, J., Graef, I., Fiorentino, D., Nourse, J., and Crabtree, G.R. (1996). The mechanism of action of cyclosporin A and FK506. Clin. Immunol. Immunopathol. *80*, S40–S45.
49. Spiekerkoetter, E., Tian, X., Cai, J., Hopper, R.K., Sudheendra, D., Li, C.G., El-Bizri, N., Sawada, H., Haghighat, R., Chan, R., et al. (2013). FK506

activates BMPR2, rescues endothelial dysfunction, and reverses pulmonary hypertension. J. Clin. Invest. *123*, 3600–3613.

50. Chaikuad, A., Alfano, I., Kerr, G., Sanvitale, C.E., Boergermann, J.H., Triffitt, J.T., von Delft, F., Knapp, S., Knaus, P., and Bullock, A.N. (2012). Structure of the bone morphogenetic protein receptor ALK2 and implications for fibrodysplasia ossificans progressiva. J. Biol. Chem. *287*, 36990–36998.

51. Hao, J., Ho, J.N., Lewis, J.A., Karim, K.A., Daniels, R.N., Gentry, P.R., Hopkins, C.R., Lindsley, C.W., and Hong, C.C. (2010). In vivo structure-activity relationship study of dorsomorphin analogues identifies selective VEGF and BMP inhibitors. ACS Chem. Biol. *5*, 245–253.

52. Peiffer, B.J., Qi, L., Ahmadi, A.R., Wang, Y., Guo, Z., Peng, H., Sun, Z., and Liu, J.O. (2019). Activation of BMP Signaling by FKBP12 Ligands Synergizes with Inhibition of CXCR4 to Accelerate Wound Healing. Cell Chem. Biol. *26*, 652–661. e654.

53. Winbanks, C.E., Weeks, K.L., Thomson, R.E., Sepulveda, P.V., Beyer, C., Qian, H., Chen, J.L., Allen, J.M., Lancaster, G.I., Febbraio, M.A., et al. (2012). Follistatin-mediated skeletal muscle hypertrophy is regulated by Smad3 and mTOR independently of myostatin. J. Cell Biol. *197*, 997–1008.

54. Pedroso, F.E., Spalding, P.B., Cheung, M.C., Yang, R., Gutierrez, J.C., Bonetto, A., Zhan, R., Chan, H.L., Namias, N., Koniaris, L.G., and Zimmers, T.A. (2012). Inflammation, organomegaly, and muscle wasting despite hyperphagia in a mouse model of burn cachexia. J Cachexia Sarcopenia Muscle *3*, 199–211.

55. Occhipinti, S., Sponton, L., Rolla, S., Caorsi, C., Novarino, A., Donadio, M., Bustreo, S., Satolli, M.A., Pecchioni, C., Marchini, C., et al. (2014). Chimeric rat/human HER2 efficiently circumvents HER2 tolerance in cancer patients. Clin. Cancer Res. *20*, 2910–2921.

56. Babic, A., Rosenthal, M.H., Sundaresan, T.K., Khalaf, N., Lee, V., Brais, L.K., Loftus, M., Caplan, L., Denning, S., Gurung, A., et al. (2023). Adipose tissue and skeletal muscle wasting precede clinical diagnosis of pancreatic cancer. Nat. Commun. *14*, 4317.

57. Mayers, J.R., Wu, C., Clish, C.B., Kraft, P., Torrence, M.E., Fiske, B.P., Yuan, C., Bao, Y., Townsend, M.K., Tworoger, S.S., et al. (2014). Elevation of circulating branched-chain amino acids is an early event in human pancreatic adenocarcinoma development. Nat Med *20*, 1193–1198.

58. Argiles, J.M., Busquets, S., and Lopez-Soriano, F.J. (2003). Cytokines in the pathogenesis of cancer cachexia. Curr. Opin. Clin. Nutr. Metab. Care *6*, 401–406.

59. Rupert, J.E., Narasimhan, A., Jengelley, D.H.A., Jiang, Y., Liu, J., Au, E., Silverman, L.M., Sandusky, G., Bonetto, A., Cao, S., et al. (2021). Tumor-derived IL-6 and trans-signaling among tumor, fat, and muscle mediate pancreatic cancer cachexia. J. Exp. Med. *218*.

60. Chen, J.L., Walton, K.L., Qian, H., Colgan, T.D., Hagg, A., Watt, M.J., Harrison, C.A., and Gregorevic, P. (2016). Differential Effects of IL6 and Activin A in the Development of Cancer-Associated Cachexia. Cancer Res. *76*, 5372–5382.

61. Mantovani, A., Allavena, P., Sica, A., and Balkwill, F. (2008). Cancer-related inflammation. Nature *454*, 436–444.

62. Zhong, W., Wu, K., Long, Z., Zhou, X., Zhong, C., Wang, S., Lai, H., Guo, Y., Lv, D., Lu, J., and Mao, X. (2022). Gut dysbiosis promotes prostate cancer progression and docetaxel resistance via activating NF-kappaB-IL6-STAT3 axis. Microbiome *10*, 94.

63. Shi, M., Zong, X., Hur, J., Birmann, B.M., Martinez-Maza, O., Epeldegui, M., Chan, A.T., Giovannucci, E.L., and Cao, Y. (2023). Circulating markers of microbial translocation and host response to bacteria with risk of colorectal cancer: a prospective, nested case-control study in men. EBioMedicine *91*, 104566.

64. Kwong, T.N.Y., Wang, X., Nakatsu, G., Chow, T.C., Tipoe, T., Dai, R.Z.W., Tsoi, K.K.K., Wong, M.C.S., Tse, G., Chan, M.T.V., et al. (2018). Association Between Bacteremia From Specific Microbes and Subsequent Diagnosis of Colorectal Cancer. Gastroenterology *155*, 383–390. e388.

65. Jarnicki, A., Putoczki, T., and Ernst, M. (2010). Stat3: linking inflammation to epithelial cancer - more than a "gut" feeling? Cell Div. *5*, 14.

66. Togashi, Y., Kogita, A., Sakamoto, H., Hayashi, H., Terashima, M., de Velasco, M.A., Sakai, K., Fujita, Y., Tomida, S., Kitano, M., et al. (2015). Activin signal promotes cancer progression and is involved in cachexia in a subset of pancreatic cancer. Cancer Lett. *356*, 819–827.

67. Thissen, J.P., and Loumaye, A. (2013). [Role of Activin A and Myostatin in cancer cachexia]. Ann. Endocrinol. *74*, 79–81.

68. Golan, T., Geva, R., Richards, D., Madhusudan, S., Lin, B.K., Wang, H.T., Walgren, R.A., and Stemmer, S.M. (2018). LY2495655, an antimyostatin antibody, in pancreatic cancer: a randomized, phase 2 trial. J Cachexia Sarcopenia Muscle *9*, 871–879.

69. Huse, M., Muir, T.W., Xu, L., Chen, Y.G., Kuriyan, J., and Massague, J. (2001). The TGF beta receptor activation process: an inhibitor- to substrate-binding switch. Mol Cell *8*, 671–682.

70. Shore, E.M., Xu, M., Feldman, G.J., Fenstermacher, D.A., Cho, T.J., Choi, I.H., Connor, J.M., Delai, P., Glaser, D.L., LeMerrer, M., et al. (2006). A recurrent mutation in the BMP type I receptor ACVR1 causes inherited and sporadic fibrodysplasia ossificans progressiva. Nat. Genet. *38*, 525–527.

71. Dumont, F.J., and Su, Q. (1996). Mechanism of action of the immunosuppressant rapamycin. Life Sci. *58*, 373–395.

72. Cetin, H., Beeson, D., Vincent, A., and Webster, R. (2020). The Structure, Function, and Physiology of the Fetal and Adult Acetylcholine Receptor in Muscle. Front. Mol. Neurosci. *13*, 581097.

73. Wyart, E., Bindels, L.B., Mina, E., Menga, A., Stanga, S., and Porporato, P.E. (2020). Cachexia, a Systemic Disease beyond Muscle Atrophy. Int. J. Mol. Sci. *21*.

74. Caan, B.J., Meyerhardt, J.A., Kroenke, C.H., Alexeeff, S., Xiao, J., Weltzien, E., Feliciano, E.C., Castillo, A.L., Quesenberry, C.P., Kwan, M.L., and Prado, C.M. (2017). Explaining the Obesity Paradox: The Association between Body Composition and Colorectal Cancer Survival (C-SCANS Study). Cancer Epidemiol. Biomarkers Prev. *26*, 1008–1015.

75. Murata, A., Amaya, K., Mochizuki, K., Sotokawa, M., Otaka, S., Tani, K., Nakagaki, S., and Ueda, T. (2018). Superior Mesenteric Artery-Pancreaticoduodenal Arcade Bypass Grafting for Repair of Inferior Pancreaticoduodenal Artery Aneurysm with Celiac Axis Occlusion. Ann Vasc Dis *11*, 153–157.

76. Avalle, L., Marino, F., Camporeale, A., Guglielmi, C., Viavattene, D., Bandini, S., Conti, L., Cimino, J., Forni, M., Zanini, C., et al. (2020). Liver-Specific siRNA-Mediated Stat3 or C3 Knockdown Improves the Outcome of Experimental Autoimmune Myocarditis. Mol Ther Methods Clin Dev *18*, 62–72.

77. Ballaro, R., Lopalco, P., Audrito, V., Beltra, M., Pin, F., Angelini, R., Costelli, P., Corcelli, A., Bonetto, A., Szeto, H.H., et al. (2021). Targeting Mitochondria by SS-31 Ameliorates the Whole Body Energy Status in Cancer- and Chemotherapy-Induced Cachexia. Cancers *13*.

78. Goodman, C.A., Mabrey, D.M., Frey, J.W., Miu, M.H., Schmidt, E.K., Pierre, P., and Hornberger, T.A. (2011). Novel insights into the regulation of skeletal muscle protein synthesis as revealed by a new nonradioactive in vivo technique. FASEB J *25*, 1028–1039.

79. Legland, D., Arganda-Carreras, I., and Andrey, P. (2016). MorphoLibJ: integrated library and plugins for mathematical morphology with ImageJ. Bioinformatics *32*, 3532–3534.

80. Shang, M., Cappellesso, F., Amorim, R., Serneels, J., Virga, F., Eelen, G., Carobbio, S., Rincon, M.Y., Maechler, P., De Bock, K., et al. (2020). Macrophage-derived glutamine boosts satellite cells and muscle regeneration. Nature *587*, 626–631.

81. Conti, L., Bolli, E., Di Lorenzo, A., Franceschi, V., Macchi, F., Riccardo, F., Ruiu, R., Russo, L., Quaglino, E., Donofrio, G., and Cavallo, F. (2020). Immunotargeting of the xCT Cystine/Glutamate Antiporter Potentiates the Efficacy of HER2-Targeted Immunotherapies in Breast Cancer. Cancer Immunol. Res. *8*, 1039–1053.

CellPress
OPEN ACCESS

82. Quaglino, E., Mastini, C., Amici, A., Marchini, C., Iezzi, M., Lanzardo, S., De Giovanni, C., Montani, M., Lollini, P.L., Masucci, G., et al. (2010). A better immune reaction to Erbb-2 tumors is elicited in mice by DNA vaccines encoding rat/human chimeric proteins. Cancer Res. *70*, 2604–2612.
83. Macagno, M., Bandini, S., Bolli, E., Bello, A., Riccardo, F., Barutello, G., Merighi, I.F., Forni, G., Lamolinara, A., Del Pizzo, F., et al. (2022). Role of ADCC, CDC, and CDCC in Vaccine-Mediated Protection against Her2 Mammary Carcinogenesis. Biomedicines *10*.
84. Jacob, J., Radkevich, O., Forni, G., Zielinski, J., Shim, D., Jones, R.F., and Wei, W.Z. (2006). Activity of DNA vaccines encoding self or heterologous Her-2/neu in Her-2 or neu transgenic mice. Cell. Immunol. *240*, 96–106.

STAR★METHODS

KEY RESOURCES TABLE

REAGENT or RESOURCE	SOURCE	IDENTIFIER
**Antibodies**		
Myoglobin	Abcam	Cat#77232; RRID:AB_1523998
FKBP12	Invitrogen	Cat#PA1-026A
FLAG Tag	Cell Signaling	Cat#8146; RRID:AB_10950495
GAPDH	Millipore	Cat#MAB374; RRID:AB_2107445
Myc-Tag	Cell Signaling	Cat#2278; RRID:AB_490778
Smad1	Cell Signaling	Cat#9743; RRID:AB_2107780
P-Smad1/5	Cell Signaling	Cat#9516; RRID:AB_491015
P-Smad1/5/9	Cell Signaling	Cat#13820; RRID:AB_2493181
STAT3	Cell Signaling	Cat#4904; RRID:AB_331269
P-STAT3	Cell Signaling	Cat#9145; RRID:AB_2491009
Vinculin	Cell Signaling	Cat#4650; RRID:AB_10559207
Puromycin	Millipore	Cat#MABE343; RRID:AB_2566826
H3K27ac	Abcam	Cat#ab4729; RRID:AB_2118291
Laminin	Santa Cruz Biotechnology	Cat#sc59854; RRID:AB_784266
Neurofilament-H (heavy)	Abcam	Cat#Ab4680; RRID:AB_304560
CD45-VioGreen	Miltenyi Biotec	Cat#130-123-900; RRID:AB_2811572
CD3-FITC	Miltenyi Biotec	Cat#130-119-135; RRID:AB_2751635
CD4-APC/Vio770	Miltenyi Biotec	Cat#130-119-134; RRID:AB_2751634
CD8-VioBlue	Miltenyi Biotec	Cat#130-123-865; RRID:AB_2811566
CD49b-PE	Miltenyi Biotec	Cat#130-102-337; RRID:AB_2660458
PD1-APC	Miltenyi Biotec	Cat#130-102-263; RRID:AB_2661365
CD11b-FITC	Miltenyi Biotec	Cat#130-113-234; RRID:AB_2733615
F4/80-PE/Vio770	Miltenyi Biotec	Cat#130-118-459; RRID:AB_2733260
Ly6C-APC/Vio770	Miltenyi Biotec	Cat#130-111-917; RRID:AB_2652804
Ly6G-VioBlue	Miltenyi Biotec	Cat#130-119-986; RRID:AB_2751964
MHCII-APC	Miltenyi Biotec	Cat#130-112-388; RRID:AB_2652906
CD11b-FITC	Miltenyi Biotec	Cat#130-113-234; RRID:AB_2652918
CD11c APC	Miltenyi Biotec	Cat#130-110-839; RRID:AB_2654709
CD69-PE/Vio770	Biolegend	Cat#104512; RRID:AB_493565
CD206-PE	Biolegend	Cat#141706; RRID:AB_10896421
PDL1-PE	BD Bioscience	Cat#558091; RRID:AB_397018
FITC-conjugated rabbit anti-mouse IgG	Dako, Milano, Italy	Cat#F313
rat anti-mouse IFN-γ (clone R4-6A2)	BD Biosciences	Cat#551216; RRID:AB_394094
IgG	Cell Signaling	Cat#2729; RRID:AB_1031062
CD16/CD32 - Fc Receptor blocker	Biolegend	Cat#101302; RRID:AB_312801
**Bacterial and virus strains**		
AAV9-shScramble	This paper	N/A
AAV9-shErfe	This paper	N/A
AAV9-shFKBP12	This paper	N/A
**Biological samples**		
Skeletal muscle biopsies from colorectal and pancreatic cancer patients and healthy donors undergoing elective surgery for non-neoplastic and non-inflammatory diseases.	Chirurgia Generale 1 - Azienda Ospedale Università Padova	N/A

(*Continued on next page*)

Continued

REAGENT or RESOURCE	SOURCE	IDENTIFIER
Chemicals, peptides, and recombinant proteins		
Activin A	R&D	Cat#338-AC
BMP6	Novus Biologicals	Cat#6325-BM
Cyclosporine A	Tocris Biosciences	Cat#1101
DMH1	Sigma	Cat#D8946
Beta-cyclodextrin	Sigma	Cat#C4767
Erythroferrone	Aviva Systems Biology	Cat#OPCA03036
FK506	Cayman Chemical	Cat#10007965
Fc hIgG2/FLAG	Laboratory of Dr. Léon Kautz	N/A
Fc-mERFE	Laboratory of Dr. Léon Kautz	N/A
ERFE globular domain (gERFE)	Laboratory of Dr. Léon Kautz	N/A
IL-6	Peprotech	Cat#216-16
Puromycin	Santa Cruz Biotechnology	Cat#sc-108071
Stattic	Abcam	Cat#120952
Torin-1	Tocris Biosciences	Cat#4247
Critical commercial assays		
Lipofectamine RNAiMAX reagent	Thermo Fisher Scientific	Cat#13778
Lipofectamine2000	Thermo Fisher Scientific	Cat#11668019
Dual-Luciferase Reporter Assay System	Promega	Cat#E1910
Pierce BCA assay	Thermo Fisher Scientific	Cat#23225
Mouse ERFE ELISA kit	Intrinsic Lifesciences	Cat#ERF-200
Protein G-Dynabeads	Life technologies	Cat#10003D
QIAquick PCR Purification Kit	Qiagen	Cat#28104
SYBR GreenER kit	Invitrogen	Cat#11784200
TRIzol reagent	Invitrogen	Cat#15596026
High Capacity cDNA Reverse Transcriptase kit	Applied Biosystems	Cat#4368814
PowerUp SYBR Green master mix	Applied Biosystems	Cat#A25742
SuperScript IV Reverse Transcriptase	Thermo Fisher Scientific	Cat#18090010
Endofree Qiagen Plasmid-Giga kit	Qiagen	Cat#12391
Experimental models: Cell lines		
C2C12 myoblasts	ATCC	Cat#CRL-1772; RRID:CVCL_0188
NIH/3T3	ATCC	Cat#CRL-1658; RRID:CVCL_0594
HEK-293T	ATCC	Cat#CRL-3216
HuH7	Laboratory of Dr. Laura Silvestri	N/A
C26	Laboratory of Dr. Nicoletta Filigheddu	N/A
Experimental models: Organisms/strains		
Female BALB/c	Charles River Laboratories	N/A
Oligonucleotides		
qPCR SYBR primers	See Table S1 for qPCR primers	N/A
siFkbp12	Qiagen	Cat#SI00171052
siAlk2	Qiagen	Cat#SI00888874
siAlk3	Qiagen	Cat#SI00929971
siCTR	Qiagen	Cat#1027310
siSTAT3	From Dr. Lidia Avalle	GenBank:NM_213659
siCTR (for siSTAT3 experiment)	From Dr. Lidia Avalle	GenBank:NM_009778
shErfe	Eurofins Genomics	TRCN0000178969
shFkbp12	Eurofins Genomics	TRCN0000012492
shScramble (from siCTR)	Eurofins Genomics	Cat#1027310
ChIP-qPCR primers	See Table S1 for ChIP-qPCR primers	N/A

(*Continued on next page*)

Continued

REAGENT or RESOURCE	SOURCE	IDENTIFIER
Recombinant DNA		
pErfe promoter_Luciferase	Vector Builder	N/A
pTK_Renilla	Promega	Cat#E2241
pGL3-(BRE)-Luciferase	Addgene	Cat#45126
pCMV6-ALK2-MYC	Laboratory of Dr. Laura Silvestri	N/A
pCMV6-ALK3-MYC	Laboratory of Dr. Laura Silvestri	N/A
pCMV6-FKBP12-FLAG	Laboratory of Dr. Laura Silvestri	N/A
pAAV_U6_shScramble	This paper	N/A
pAAV_U6_shERFE	This paper	N/A
pAAV_U6_shFKBP12	This paper	N/A
pVAX1	Invitrogen	Cat#V26020
pRHuT	Laboratory of Dr. Laura Conti	N/A
Software and algorithms		
Prism GraphPad 6	Prism	https://www.graphpad.com/scientific-software/prism/
ImageJ	Fiji Image	https://imagej.net/Fiji
Adobe Illustrator 2022	Adobe	https://www.adobe.com/it/products/illustrator.html
Biorender	Biorender.com	https://www.biorender.com/

RESOURCE AVAILABILITY

Lead contact

Requests for resources and reagents, or any additional information should be directed to the lead contact, Dr. Paolo Ettore Porporato (paolo.porporato@unito.it).

Materials availability

Plasmids generated in this study will be available on request through completion of a material transfer agreement.

Data and code availability

Any additional information required to reanalyze the data reported in this paper, or any sharing of data of this study is available from the lead contact upon request. This paper does not report original code.

EXPERIMENTAL MODEL AND SUBJECT DETAILS

Human skeletal muscles biopsies

The study enrolled patients (age >18 years) with colon or pancreatic cancer surgically treated at the General Surgery 1, Padova University Hospital (Padova, Italy) from 2016 to 2021. Cancer patients were classified as cachectic in cases of >5% weight loss in the 6 months preceding surgery, >2% weight loss with either body mass index (BMI) < 20 or low muscle mass defined by the skeletal muscle index (SMI) cut-offs.[74] SMI values were quantified using the preoperative CT scans as previously described.[14] The study also enrolled control, healthy donors undergoing elective surgery for non-neoplastic and non-inflammatory diseases. Patients with presence of active inflammatory or infective diseases, known myopathies, or viral infections were excluded. All patients joined the protocol according to the guidelines of the Declaration of Helsinki and the research project has been approved by Ethical Committee for Clinical Experimentation of Provincia di Padova (protocol number 3674/AO/15). Written informed consent was obtained from participants. The biopsies were collected during elective surgery within 30 min of the start of the surgery by cold section of a rectus abdominal fragment of about 0.5–1 cm. The fragment was immediately frozen and conserved in liquid nitrogen for gene expression analysis.

Animals

All animal experiments were authorized by the Italian Ministry of Health and carried out according to the European Community guiding principles in the care and use of animals.

All mice were 6–8 weeks old female BALB/c, purchased from Charles River Laboratories and housed in a pathogen-free environment and kept food and water *ad libitum*.

Cell lines

C2C12 myoblasts (CRL-1772), NIH/3T3 (CRL-1658), HEK-293T (CRL-3216), cells were purchased from ATCC, while HuH7 cells were provided by Dr. Laura Silvestri and colon-26 (C26) cells were kindly gifted by Dr. Nicoletta Filigheddu (Università del Piemonte Orientale). All cells were cultured in DMEM/10% FBS. For C2C12 myoblasts differentiation into myotubes, when full confluency is reached, the medium is switched to 2% horse serum (HS) DMEM for 4 days.

METHOD DETAILS

Cell culture

Conditioned medium (CM) was prepared as previously described.[10] Briefly, cancer cells were grown to high confluency, then conditioned in serum-free DMEM for 24h, medium was harvested and centrifuged at 500g for 10min. Supernatant was subsequently used as conditioned medium.

Myotubes were treated with Activin A (R&D #338-AC), BMP6 (Novus Biologicals #6325-BM), cyclosporine A (Tocris Biosciences #1101), DMH1 (Sigma #D8946) and beta-cyclodextrin (Sigma #C4767), Erythroferrone (Aviva Systems Biology OPCA03036), FK506 (Cayman Chemical 10007965), Fc hIgG2/FLAG (CTR Fc), Fc hIgG2/FLAG-ERFE and ERFE globular domain (gERFE) were kindly provided by Prof. Leon Kautz (IRSD, Toulouse), IL-6 (Peprotech #216-16), puromycin (Santa Cruz Biotechnology sc-108071), stattic (Abcam #120952), Torin-1(Tocris Biosciences #4247).

For *in vitro* immunofluorescence, C2C12 myoblasts were seeded in a 96-well plate (Ibidi) and differentiated into myotubes. Once treated, cells were fixed in 4% PFA at room temperature and then permeabilized with 0.1% Triton X-100. Nonspecific binding was blocked by incubation of permeabilized cells in 1% BSA for 1h at room temperature. Cells were then incubated overnight at 4°C with a primary antibody against myoglobin (Abcam #77232) diluted 1:400 in 1% BSA. A secondary antibody conjugated with Alexa Fluor 488 (Thermo Fisher) was applied for 1 h at room temperature. Pictures were acquired with a confocal microscope (Leica SP8).

Myotube diameter measurement

For Myotube diameter quantification, pictures of myotubes were taken 24h after treatment with phase contrast microscopy (Zeiss) at 20x magnification, and myotube diameter was measured using the software ImageJ as previously described.[75]

siRNA-induced knockdown

C2C12 myotubes were transfected with siRNAs at day three of differentiation using Lipofectamine RNAiMAX reagent (Thermo Fisher Scientific, #13778). Briefly, in a 12-well plate, the medium was replaced with 200 μL fresh medium, transfection mix was prepared (2 × 25μL Opti-MEM, Thermo Fisher Scientific, #1985047; 2 μL RNAiMAX; 50pmol siRNA) and carefully added. After 4-6h 1mL of medium was added and myotubes were analyzed 72h post-transfection. siRNA sequences are reported in the key resources table.

NIH/3T3 were knocked-down with siSTAT3 or with siCTR (1 μg/mL) for 72 h, as previously described.[76]

Luciferase assay

For Erfe promoter-Luciferase reporter assay, Erfe promoter was cloned in a luciferase vector and purchased on Vector Builder. NIH/3T3 cells were co-transfected with 1 μg of DNA (ratio 1:6 Renilla:Firefly) using Lipofectamine2000 (Thermo Fisher Scientific, #11668019). After 16h, the transfection mix is aspired and substituted with siSTAT3 or siCTR mix to induce STAT3 knockdown. 50% C26 CM or IL-6 (100 ng/mL) were then added to the cells 72h post plasmid transfection, for 16h. Dual-Luciferase Reporter Assay System (Promega) was then performed following the manufacturer's instructions.

C2C12 myoblasts were seeded into a 48-well plate and co-transfected with pGL3-BMP responsive element (BRE)-Luc plasmid and TK Renilla. Co-transfection was performed with 800 ng of DNA/well at a 1:6 ratio of Renilla:Firefly using Lipofectamine2000. Transfection mix was added to each well containing 100 μL of fresh medium. 4-6 h post-transfection 500 μL of fresh medium was added into each well. The next day, cells were serum starved for 4h and subsequently treated with 30 ng/ml BMP6 and 10 μg/ml FK506 in 2% FBS medium. Luminescence was measured using Dual-Luciferase Reporter Assay System (Promega) after 5 h of treatment.

Western blotting

Crushed skeletal muscle samples or C2C12 myotubes were lysed in RIPA lysis buffer (150 mM NaCl, 50 mM Tris-HCl, 0.5% sodium deoxycholate, 1.0% Triton X-100, 0.1% SDS, and 1mM EDTA) supplemented with protease and phosphatase inhibitor cocktail (Roche). Protein concentration was determined using BCA assay (Thermo Fisher Scientific). 15 to 30 μg of proteins from cell lysates were loaded for SDS-PAGE and then transferred to PVDF membrane prior to immunoblotting analysis. Blots were probed with the following primary antibodies: FKBP12 (Invitrogen, PA1-026A), FLAG Tag (Cell Signaling, #8146), GAPDH (Millipore, MAB374), Myc-Tag (Cell Signaling, #2278), Smad1 (Cell Signaling, #9743), P-Smad1/5 (Cell Signaling, #13820T), pSmad1/5/9 (Cell Signaling, #13820), STAT3 (Cell Signaling, #4904), pSTAT3 (Cell Signaling, #9145), vinculin (Cell Signaling, #4650), puromycin (Millipore, MABE343).

In vitro co-immunoprecipitation (Co-IP)

The pCMV6-ALK2-MYC, the pCMV6-ALK3-MYC and the pCMV6-FKBP12-FLAG expressing vectors were kindly provided by Prof. Laura Silvestri (San Raffaele Scientific Institute, Milan).

HuH7 cells were lipofected with FKBP12-FLAG and ALK2-MYC or ALK3-MYC using Lipofectamine 2000 (Thermo Fisher Scientific, #11668019) according to manufacturer's instructions. Briefly, HuH7 cells were transfected with 20 μg DNA with 1:1 ratio (ALK:FKBP12 or ALK:pCMV6 empty vector) in antibiotic-free medium. 16 h after lipofection, the medium is changed with fresh DMEM/10%FBS.

HEK-293T cells were transfected in HEPES and CaCl2 with 5 μg DNA with 1:1 ratio (ALK:FKBP12 or ALK:pCMV6 empty vector) and 16h later, the medium is refreshed with DMEM/10%FBS.

The next day, HuH7 or 293T cells are starved in DMEM/2%FBS for 3h and treated with FK506 for 1h. Cells are subsequently washed and pelleted in ice-cold PBS. Pellets are lysed in NET buffer (50mM NaCl, 5 mM EDTA, 10 mM Tris pH 7,4, 1% Triton X-100) and protease inhibitors. 500 μg of proteins are incubated with the anti-FLAG M2 affinity gel (Sigma Aldrich) at 4°C for 2 h. After gel washing, samples were eluted with 18μL of Laemmli sample buffer (without β-mercaptoethanol) and incubated at 95°C for 5 min. After centrifugation, β-mercaptoethanol was added to supernatants and SDS-PAGE was performed.

Animal experimentation

Adeno-associated virus (AAV) AAV9-shRNA delivery was performed in 8 weeks old female BALB/c mice under inhalation of isoflurane in medical oxygen. 10^{11} AAV9-shRNA or AAV9-shCTR viral particles were resuspended in PBS for a final volume of 50uL. Using the Hamilton (PB600-1) Repeating Dispensers (1uL), 10 injections of 5uL were performed in the gastrocnemii of the mice. AAV9-shCTR injections were executed in the contralateral gastrocnemius, and the two constructs were equally side-exchanged within the experimental groups.

Four weeks after AAV9 delivery, C26 murine adenocarcinoma cell suspension (750.000 cells per mouse) was inoculated subcutaneously into the flank and euthanized after 11 days

Concerning all FK506 (Cayman Chemical 10007965) *in vivo* administration, FK506 was dissolved in DMSO (5 mg/mL) and further diluted in glucose-water (5%) to daily administer 0.02 mg/kg in 100uL of volume, through oral gavage. All mice were daily treated either with vehicle DMSO buffer glucose (5%) or with FK506.

For RHuT immunization, 6–8 weeks old male or female BALB/c mice were pre-treated for 1 week with FK506 or vehicle before the first dose of vaccination.

In C26 experiments, all mice underwent one day of pre-conditioning before C26 inoculation. The body weight of the mice was measured every 3 days, while the maximal grip strength was measured every 4 days with BIOSEB (BIO-GS3) instrument. All mice were anesthetized and sacrificed at day 11 post-inoculation. Fresh blood was collected through intracardiac puncture and serum was extracted by centrifuging clotted blood at 2,000g for 15 min at 4°C. All gastrocnemii (Gx), quadriceps (Qd), tibialis anterior (TA), extensor digitorum longus (EDL), white fat deposits, livers, spleens and tumors were freshly isolated, weighted and normalized to the respective tibial length, measured with a caliper.

Eythroferrone enzyme-linked immunosorbent assay (ELISA)

Serum protein levels of erythroferrone were measured with Intrinsic Lifesciences mouse ERFE ELISA kit (ERF-200) according to manufacturer's instructions.

Protein synthesis measurement with SuNSET assay

To assess protein synthesis in skeletal muscles *in vitro* and *in vivo*, SuNSET assay was performed as previously described.[77,78]

Concisely, C2C12 myotubes were treated with C26 CM or Activin A in DMEM/2%HS for 24h. For the last 4h of treatment, cells were supplemented with puromycin (1 μM) and subsequently lysed in RIPA with protease and phosphatase inhibitors. Any kD SDS-PAGE was then performed prior to immunoblotting with puromycin antibody.

For the *in vivo* measurements of protein synthesis (IV-SuNSET), all mice received an intraperitoneal injection of 0.040 μmol/g of puromycin dissolved in 100 μL of PBS. Subsequently, they were anesthetized and sacrificed in order to snap-freeze in liquid N2 all muscles exactly at 30 min after puromycin injection. Whole protein lysates in RIPA with protease and phosphatase inhibitors were then processed for SDS-PAGE and immunoblotting as described before.

C2C12 myotubes and skeletal muscles chromatin immunoprecipitation (ChIP-qPCR)

Three fully confluent 10 cm dishes of C2C12 myotubes per condition (untreated and pure C26 CM for pSTAT3, STAT3, H3K27ac, IgG) were crosslinked with 1% formaldehyde for 10 min at room temperature. Formaldehyde was quenched by 0.125 mM glycine for 5 min at room temperature. Crosslinked cells were quickly washed with cold PBS and collected in cold PBS.

Nuclear extracts were prepared by using isotonic buffer. Briefly, C2C12 myotubes were lysed in isotonic buffer containing 20 mM HEPES pH 7.5, 100 mM NaCl, 250 mM Sucrose, 5 mM MgCl2, 5 mM ZnCl2 supplemented with 1% NP40. Cell pellets were resuspended in SDS ChIP lysis buffer containing 50mM Tris-HCl pH 8.0, 10 mM EDTA, 0.5% SDS and protease inhibitors. Chromatin was sheared to an average DNA fragment length of 200–500 bp using Picoruptor sonicator (Diagenode) (12 cycles, 30s on/off). Cell lysates were diluted five times with the ChIP dilution buffer lacking SDS and composed by 17 mM Tris-HCl pH 8, 1% Triton X-100, 1.2 mM EDTA, 170 mM NaCl to a final concentration of 0.1% SDS and used for immunoprecipitation with 4 μg of anti-Stat3 (phospho Y705) antibody (Cell Signaling, #9145), anti-H3K27ac antibody (Abcam, ab4729), and an unspecific normal rabbit IgG as a control. After overnight incubation of the chromatin with the antibodies at 4°C, the immunocomplexeswere captured with 10 μL of Protein G-Dynabeads (Life technologies) for further 2 h at 4°C.

For Stat3 ChIP, the protein G-bound immunocomplexes were washed four times with buffer containing 50mM HEPES pH 7.6, 500mM LiCl, 1 mM EDTA, 1% NP-40, 0.7% Sodium Deoxycholate and protease inhibitors, while for H3K27ac ChIP were washed with the following buffers: low salt wash buffer containing 20 mM Tris-HCl pH 8.0, 0.1% SDS, 1% Triton X-100, 2 mM EDTA, 150 mM NaCl; high salt wash buffer containing 20 mM Tris-HCl pH 8.0, 0.1% SDS, 1% Triton X-100, 2 mM EDTA, 500 mM NaCl; LiCl wash buffer containing 10 mM Tris-HCl pH 8.0, 1% Sodium Deoxycholate, 250 mM LiCl, 1 mM EDTA, 1% NP40 and protease inhibitors. Immunocomplexes were eluted from the beads and crosslinking was reversed by incubation for overnight at 65°C with TE buffer containing 1% SDS. DNA from immunoprecipitated samples as well as DNA from 10% input was purified by using QIAquick PCR Purification Kit (Qiagen), according to the manufacturer's instruction.The Stat3 ChIP and the H3K27ac ChIP were analyzed by ChIP-qPCR using SYBR GreenER kit (Invitrogen) on target genomic regions (Table S1). qPCR reactions were performed on a Rotor-Gene Q 2plex HRM Platform (Qiagen, 9001560). The data are expressed as a percentage of the DNA Input.

For *in vivo* chromatin immunoprecipitation, two entire frozen quadriceps per condition (CTR and C26 for pSTAT3, H3K27ac, IgG) were homogenized with a Dounce homogenizer in 1,200 μL nuclear extraction buffer composed by 10 mM Tris HCl, 10 mM NaCl, 3 mM $MgCl_2$, 10 mg/mL BSA, 0.1% IGEPAL, 0.5 mM DTT and protease inhibitors. Afterward, 37% formaldehyde was added to the lysates to reach a final concentration of 1%, and incubated at room temperature for 10 min, tapping occasionally to mix. The crosslinking reaction was interrupted by adding 75 μL of 1.25 M glycine on ice for 5 min. Samples were centrifuged at 1000*g*, 4°C for 10 min. Cell pellets were resuspended in SDS ChIP lysis buffer containing 50 mM Tris-HCl pH 8.0, 10 mM EDTA, 0.3% SDS and protease inhibitors. Chromatin was sheared to an average DNA fragment length of 500 bp using Picoruptor sonicator (Diagenode) (15 cycles, 30 s on/off). After sonication, the samples were processed as *in vitro* ChIP-qPCR.

Histology

Extracted gastrocnemii were immediately frozen in isopentane cooled in liquid nitrogen and stored at −80°C. Transversal sections of 10μm were cut at the midbelly with a cryostat. Cross sectional area (CSA) was determined by sections fixation for 10 min in PFA 4% PBS, then blocking with Triton X-100 0.1%, BSA 3% in PBS before incubating with primary antibodies against laminin (Santa Cruz Biotechnology sc-59854), followed by incubation with corresponding secondary antibodies (Alexa Fluor 488) and DAPI (Abcam 228549). Pictures were taken as mosaic of the full section with a confocal microscope (Leica SP5) and fiber areas were measured with MorphoLibJ plugin for ImageJ.[79]

Neuromuscular junction analysis

Extensor digitorum longus (EDL) muscles were immediately fixed in PFA 4% PBS for 10 min and placed in cold PBS. The full muscles were stained with fluorescently tagged α-bungarotoxin - Alexa 488 (Thermo Fisher B13422) for 90 min 1:500 in PBS at room temperature. The whole muscles were mounted and pictured with fluorescence confocal microscopy (Leica SP5).

Each post-synaptic NMJ has been acquired through 1 μm thick z stack, and voxel were automatically quantified with ImageJ software.

For denervation quantification, fixed EDL muscles were permeabilized for 2 h at room temperature in 2% Triton X-100 in PBS, then blocked in 4% BSA - 1% Triton X-100 in PBS. The primary antibody anti-neurofilament-H (heavy) (Abcam #Ab4680) was incubated 1:1000 in blocking buffer at 4°C overnight. 3 x 20′ PBS washes were followed by secondary antibody and fluorescently tagged α-bungarotoxin - Alexa 488 (Thermo Fisher B13422) incubation and whole mount. Pictures were acquired with fluorescence confocal microscopy (Leica SP8). Denervation was measured evaluating 60 to 90 NMJs per mouse (with a total of three mice per treatment group). Our scoring system, ranged from 0 to 2: 0 for complete denervation, 1 for partial denervation, and 2 for full innervation.

RNA isolation and quantitative PCR (qPCR)

Total RNA was isolated from snap-frozen tissue samples using TRIzol reagent (Invitrogen) according to the manufacturer's guidelines. 0.5μg of total RNA was reverse transcribed using the High Capacity cDNA Reverse Transcriptase kit (Applied Biosystems) following the manufacturer's instructions. cDNA was analyzed by Real Time Quantitative PCR (ABI PRISM 7900HT FAST or Quant Studio 6 Real-Time PCR system, Applied Biosystems) using the PowerUp SYBR Green master mix (Applied Biosystems #A25742) and primers were designed according to Harvard validated PrimerBank. Relative mRNA levels were calculated using the 2-ΔΔCT method and normalized to GAPDH mRNA. For human muscle biopsies, total RNA was extracted from approximately 20 mg of rectus abdominal muscle using TRIzol (Invitrogen). 1 μg of RNA was reverse transcribed using the SuperScript IV Reverse Transcriptase (Thermo Fisher Scientific). Gene expression was analyzed by qRT-PCR (Quant Studio 5 Real-Time PCR system, Applied Biosystems) using the PowerUp SYBR Green Master Mix (Applied Biosystems #A25742). Data were normalized to beta-actin gene expression. All RT-qPCR primers are listed in Table S1.

Adeno-associated virus (AAV) shRNA cloning

For the *in vivo* adeno-associated virus (AAV9-shRNA) delivery, short hairpin sequences targeting ERFE (shERFE - TRCN0000178969), FKBP12 (shFKBP12 - TRCN0000012492) or scramble sequence (shCTR) were cloned in the AAV plasmid and assembled in viral particles as previously described.[78,80]

FACS

Fresh primary C26 tumor specimens of 8–10 mm mean diameter were finely minced with blades and digested by incubation with 1 mg/mL collagenase IV (Sigma Aldrich) in RPMI-1640 at 37°C for 1 h in an orbital shaker. After washing in RPMI-1640 with 10% FBS, the cell suspension was passed through a 70 μm pore cell strainer, centrifuged at 1400 rpm for 10 min and incubated in erythrocyte lysing buffer (155 mM NH_4Cl, 15.8 mM Na_2CO_3, 1 mM EDTA, pH 7.3) for 10 min at room temperature. After washing in RPMI-1640 with 10% FBS, 10^6 cells were collected, re-suspended in PBS, and treated with Fc-receptor blocker (anti-CD16/CD32 antibody, #101302, Biolegend). Splenocytes were collected by smashing spleens from vaccinated mice on a 40 μm pore cell strainer, centrifuging the resulting cells at 1,400 rpm for 10 min and incubating them in erythrocyte lysing buffer, followed by treatment with Fc-receptor blocker. 50 μL heparinized blood collected by intracardiac injection were incubated with erythrocyte lysing buffer, washed and incubated with Fc-receptor blocker. After Fc-receptor blocking, all samples were stained for 30 min at 4°C with the following antibodies, as reported previously[81]:

anti-CD45-VioGreen (#130-123-900), anti-CD3-FITC (#130-119-135), anti-CD4-APC/Vio770 (#130-119-134), anti-CD8-VioBlue (#130-123-865), anti-CD49b-PE (#130-102-337), anti-PD1-APC (#130-102-263), anti-CD11b-FITC (#130-113-234), anti-F4/80-PE/Vio770 (#130-118-459), anti-Ly6C-APC/Vio770 (#130-111-917), anti-Ly6G-VioBlue (#130-119-986), anti-MHCII-APC (#130-112-388), anti-CD11b-FITC (#130-113-234), anti-CD11c APC (#130-110-839) (all from Miltenyi Biotec) anti-CD69-PE/Vio770 (#104512, Biolegend), anti-CD206-PE (#141706, Biolegend) and anti-PDL1-PE (# 558091, BD Bioscience). Samples were acquired on BD-FACSVerse and cell populations (NK: $CD3^-$ $CD49b^+$; NKT: $CD3^+$ $CD49b^+$; $CD4^+$ T cells: $CD3^+$ $CD49b^-$ $CD4^+$; $CD8^+$ T cells: $CD3^+$ $CD49b^-$ $CD8^+$; DC: CD11b- CD11c+; macrophages: $CD11b^+$ $F4/80^+$; *m*-MDSC: $CD11b^+$ $F4/80^-$ $Ly6G^-$ $Ly6C^{high}$; gMDSC: $CD11b^+$ $F4/80^-$ $Ly6G^+$ $Ly6C^{dim/neg}$) analyzed with FlowJO10.5.3 and reported as percentage on the $CD45^+$ leukocyte population, following doublets and dead cells elimination.

Vaccine preparation and mouse immunization

A plasmid coding for chimeric rat and human ErbB2 extracellular and transmembrane domains (RHuT)[82] and the corresponding control empty plasmid pVAX1 (Invitrogen, Monza, Italy) were amplified and then purified using the Endofree Qiagen Plasmid-Giga (Qiagen Inc., Cjatsworth, CA) following manufacturer's instructions. Vaccination was performed by injecting 50 μg of DNA diluted in 20 μL of 0.9% NaCl into the quadriceps muscle of anesthetized mice. Immediately after the vaccine injection, the muscle was electroporated by using an array needle electrode connected to an electroporator (Cliniporator, IGEA, Carpi, Italy). Two 25-ms *trans*-cutaneous low voltage electric pulses with an amplitude of 150 V spaced by a 300-ms interval were applied.[83] Vaccination was repeated 14 days later. Two weeks after, mice were culled and sera and spleens were collected.

Antibody response

Blood samples were collected 14 days after the last vaccination and serum obtained by centrifugation. The concentration of anti-rat Her2 antibodies was determined by flow cytometry as the ability of diluted sera (1:200) to bind 3T3/NKB cells (BALB/c 3T3 NKB, expressing the rat ErbB2, H-$2K^d$, and B7.1 molecules),[84] which were a generous gift from Dr. Wei-ZenWei (Karmanos Cancer Institute, Detroit, MI, USA). A FITC-conjugated rabbit anti-mouse IgG antibody (F313, Dako, Milano, Italy) was used to detect the bound primary antibodies. Flow cytometry was performed with a BD-FACSVerse and samples were analyzed with FlowJO10.5.3, and antibody titers reported as mean fluorescence intensity (MFI).

IFN-γ enzyme-linked immunospot (ELISpot) assay

Splenocytes from vaccinated mice were plated at 1×10^6/well into 96-well HTS IP plates (Millipore) precoated with 5 μg/mL of rat anti-mouse IFN-γ (clone R4-6A2, BD Biosciences). Cells were then stimulated with 15 μg/mL of rat ErbB2 immunodominant peptide (TYVPANASL) peptide for 16 h in the incubator, or with concanavalin A (2 μg/ml) or medium alone as positive and negative controls, respectively. Images of the wells were acquired, and IFN-γ spots enumerated, with a microplate reader, along with a computer-assisted image analysis system (Immunospot; CTL Europe, Bonn, Germany), as previously described.[82]

QUANTIFICATION AND STATISTICAL ANALYSIS

All graphs show mean ± SEM and were analyzed with GraphPad Prism (version 6.0, GraphPad Software). Detailed statistical tests are reported in figure legends, where n represents the total number of independent experiments. For *in vivo* data, n is referred to the number of mice per group.

Statistical significance was tested with unpaired t-test when two groups of data were compared, with one-way ANOVA followed by Sidak's multiple comparison test when more than two groups were analyzed. Two-way ANOVA followed by Sidak's multiple comparison test was performed when two variables were analyzed. When relevant, one sample t-test was performed. For human biopsies analysis non-parametric Krustal-Wallis test followed by Benjamini, Krieger and Yekutieli multiple comparison test was performed. Significance was defined as *$p < 0.05$, **$p < 0.01$, and ***$p < 0.001$. The illustrations were created on Biorender.com and all figures were assembled using Adobe Illustrator (AI, 2022).

Cell Reports Medicine, Volume *4*

Supplemental information

FK506 bypasses the effect of erythroferrone

in cancer cachexia skeletal muscle atrophy

Erica Mina, Elisabeth Wyart, Roberta Sartori, Elia Angelino, Ivan Zaggia, Valentina Rausch, Mara Maldotti, Alessia Pagani, Myriam Y. Hsu, Alberto Friziero, Cosimo Sperti, Alessio Menga, Andrea Graziani, Emilio Hirsch, Salvatore Oliviero, Marco Sandri, Laura Conti, Léon Kautz, Laura Silvestri, and Paolo E. Porporato

Supplementary Figure 1

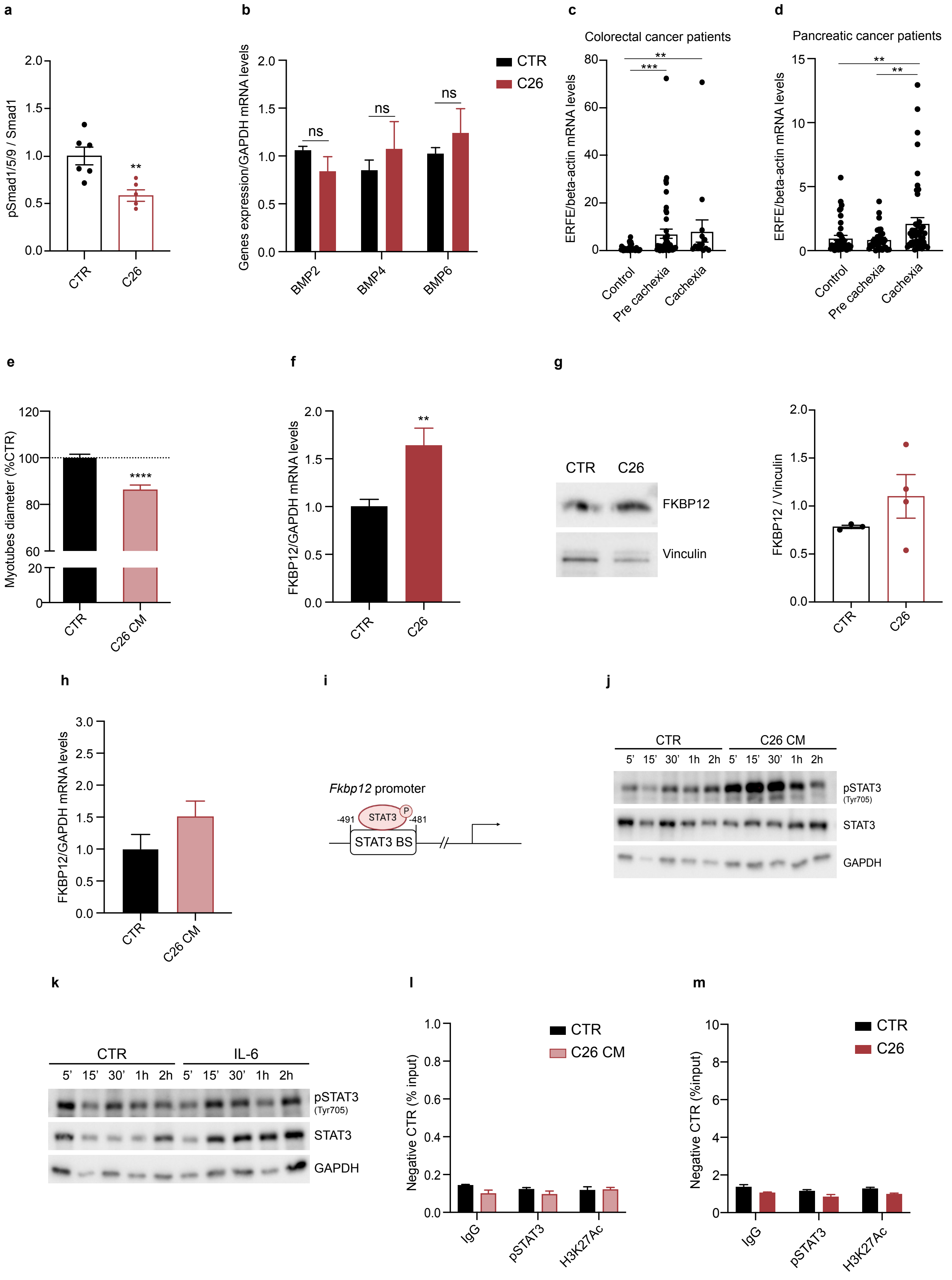

Supplemental information

Figure S1: The BMP-scavenger erythroferrone (ERFE) is upregulated in cachexia through STAT3 activation. Related to Fig. 1.

a) Densiometric quantification of the immunoblot of pSmad1/5/8 and Smad1 in the quadriceps of control and C26 tumor-bearing mice. (n=5/6) **b)** Expression levels of BMP2 (n=4/5), BMP4 (n=4) and BMP6 (n=9), in the gastrocnemii of control and C26 tumor-bearing mice. **c)** Expression levels of *ERFE* relative to beta actin in human skeletal muscle biopsies from pre-cachectic and cachectic colorectal cancer patients compared to control individuals (Ctr, n=41; pre-cachectic, n=45; cachectic, n=15; control vs pre-cachexia q value=0.0001; control vs cachexia q value=0.0016). Values are reported as fold change over control group. **d)** Expression levels of *ERFE* relative to beta actin in human skeletal muscle biopsies from pre-cachectic and cachectic pancreatic cancer patients compared to control individuals (Ctr, n=41; pre-cachectic, n=32; cachectic, n=48; control vs cachexia q value=0.0022; pre-cachexia vs cachexia q value=0.0091;). Values are reported as fold change over control group**. e)** C2C12 myotubes diameter treated with 20% C26 CM for 24h. (n=5) **f)** mRNA levels of FKBP12 relative to GAPDH in the gastrocnemii of C26 tumor-bearing mice and relative control mice 11 days post cancer cells inoculation. (n=8) **g)** Immunoblot and densiometric quantification of FKBP12 in the quadriceps of control and C26 tumor-bearing mice. (n=3/4) **h)** Expression levels of FKBP12 in C2C12 myotubes treated for 48h with 20% C26 CM (n=6). **i)** Putative STAT3 binding site on *Fkbp12* promoter. **j-k)** Time course and immunoblot of phosphorylated STAT3 (Tyr705 – pSTAT3) and total STAT3 in C2C12 myotubes serum-starved for 2h and treated with pure C26 CM **(j)** (n=2) or IL-6 (100ng/mL) **(k)** or serum-free DMEM (CTR) at different timepoints. (n=2) **l-m)** ChIP-qPCR on a negative control region characterized by the absence of pSTAT3 and H3K27Ac in C2C12 myotubes treated with C26 CM (**l**) and *in vivo* skeletal muscles (**m**) compared to the control (each condition represents the pooling of 4 quadriceps). (n=2)

Data information: Statistical significance was tested with unpaired two-tailed student's t-test in graphs a, b, e-h; with Kruskal-Wallis test followed by Benjamini, Krieger and Yekutieli multiple comparison test in graphs c, d; with two-way ANOVA followed by Sidak's multiple comparison test in graphs l-m.

Supplementary Figure 2

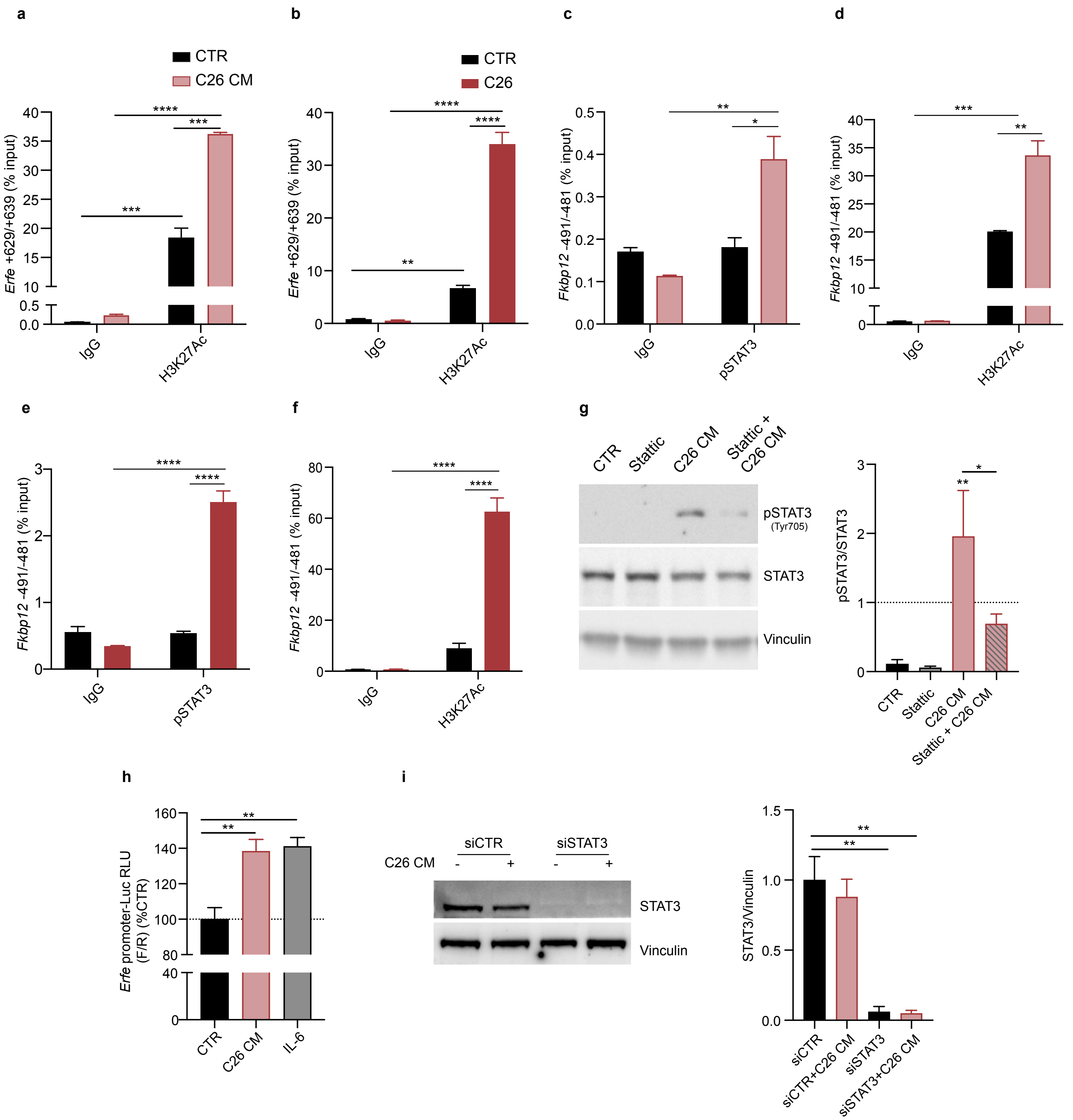

Figure S2: The BMP-scavenger erythroferrone (ERFE) is upregulated in cachexia through STAT3 activation. Related to Fig. 1.

a) ChIP-qPCR of acetylated H3K27 of *Erfe* promoter on TSS (+629/+639 bp) in C2C12 myotubes treated with C26 CM compared to control cells. (n=2) **b)** *In vivo* ChIP-qPCR of acetylated H3K27 of *Erfe* promoter on TSS (+629/+639 bp). ChIP-qPCR assay was performed by pooling 4 quadriceps per condition for control or C26 tumor-bearing mice. (n=2). **c-d)** *In vitro* ChIP-qPCR of pSTAT3 binding *Fkbp12* promoter on TSS (-491/-481) (**c**). The binding sites also carried an enrichment of the histone modification-H3K27ac as indication of open chromatin (**d**). ChIP-qPCR assay was performed on C2C12 myotubes serum-starved for 2h and treated for 30' with pure C26 CM. (n=2) **e-f)** *In vivo* ChIP-qPCR of pSTAT3 binding *Fkbp12* promoter on TSS (-491/-481) (**e**). The pSTAT3 binding sites also carried the histone modification-H3K27ac (**f**). ChIP-qPCR assay was performed by pooling 4 quadriceps per condition for control or C26 tumor-bearing mice. (n=2) **g)** Representative immunoblot of pSTAT3 (Tyr705) and relative densitometric quantification of C2C12 myotubes treated with Stattic 5uM and/or pure C26 CM for 30', previously serum-starved for 2h. (n=3) **h)** Luminescence signal (firefly/renilla) of NIH/3T3 cells transfected with *Erfe* promoter-Luc reporter vector and treated with 50% C26 CM or IL-6 (100ng/mL) overnight (n=4/6). **i)** Representative STAT3 immunoblot and relative densitometric quantification of NIH/3T3 cells transfected as in Fig. 1m (n=2).

Data information: Statistical significance was tested with unpaired two-tailed student's t-test in graph h; with two-way ANOVA followed by Sidak's multiple comparison test in graphs a-f, and with one-way ANOVA followed by Sidak's multiple comparison test in graph g, i.

Supplementary Figure 3

a

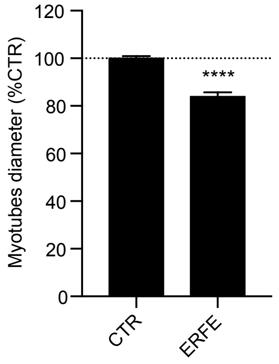

b

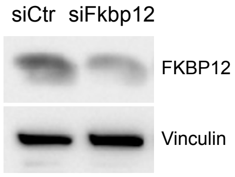

Figure S3: Muscle-specific knockdown of BMP inhibitors curbs muscle wasting in C26 tumor-bearing mice. Related to Fig. 2.

a) Quantification of C2C12 myotubes treated with murine recombinant ERFE (1ug/mL) for 24h. (n=5/7) **b)** Immunoblotting for *Fkbp12* knock-down in myotubes transfected with siRNA for *Fkbp12* or scramble sequence (siCtr) for 48h (n=2).

Data information: Statistical significance was tested with unpaired two-tailed student's t-test in graph a.

Supplementary Figure 4

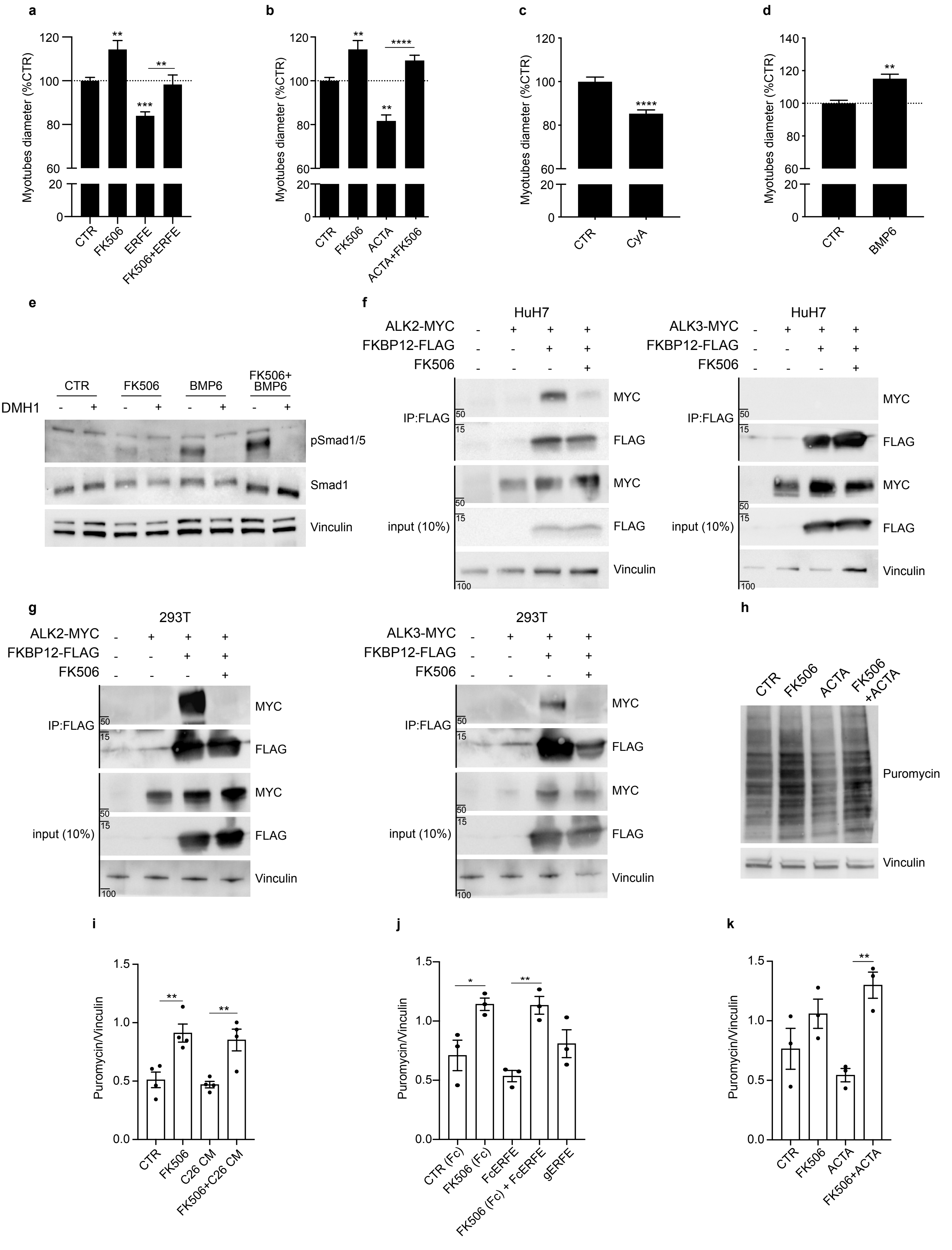

Figure S4: FK506 bypasses BMP-pathway inhibition by activating pSmad1/5/8 through FKBP12 binding. Related to Fig. 3.

a-d) Myotubes diameters quantified at 24h after treatment with FK506 (1ug/mL), ERFE (1ug/mL) (**a**), Activin A (ACTA) (1ng/mL) (**b**), cyclosporine A (CyA) (1uM) (**c**), or BMP6 (1ng/mL) (**d**) in differentiation medium. (n=3/5) **e)** Immunoblot for pSmad1/5/8 and Smad1 of C2C12 myotubes serum-starved for 2h and treated with FK506 (1ug/mL), BMP6 (30ng/mL) with or without DMH1 (500nM) for 1h in serum-free DMEM. (n=3) **f-g)** HuH7 (**f**) and HEK-293T (**g**) cells were transfected with FKBP12-FLAG and ALK2-MYC or ALK3-MYC constructs for 48h. Afterwards, cells were switched to low serum medium (DMEM 2%FBS) for 3h and treated for 1h with FK506 (10ug/mL for HEK-293T and 1ug/mL for HuH7). Protein lysates were then immunoprecipitated using FLAG-tagged resin and immunoblotting was performed to detect the interaction between FKBP12 and ALK2 or ALK3. (n=4) **h)** *In vitro* SuNSET assay performed in C2C12 myotubes treated with FK506 (1ug/mL) and Activin A (ACTA) (1ng/mL) for 24h in differentiation medium. For the last 4h of treatment, puromycin (1uM) was added to the cells. (n=3) **i-k)** Densiometric quantifications of *in vitro* SuNSET assays performed in C2C12 myotubes treated with FK506, C26 CM (**i**), FcERFE (**j**) or ACTA (**k**). (n=3/4)

Data information: Statistical significance was tested with one-way ANOVA followed by Sidak's multiple comparison test in graph a, b, i-k; with unpaired two-tailed student's t-test in graphs c, d.

Supplementary Figure 5

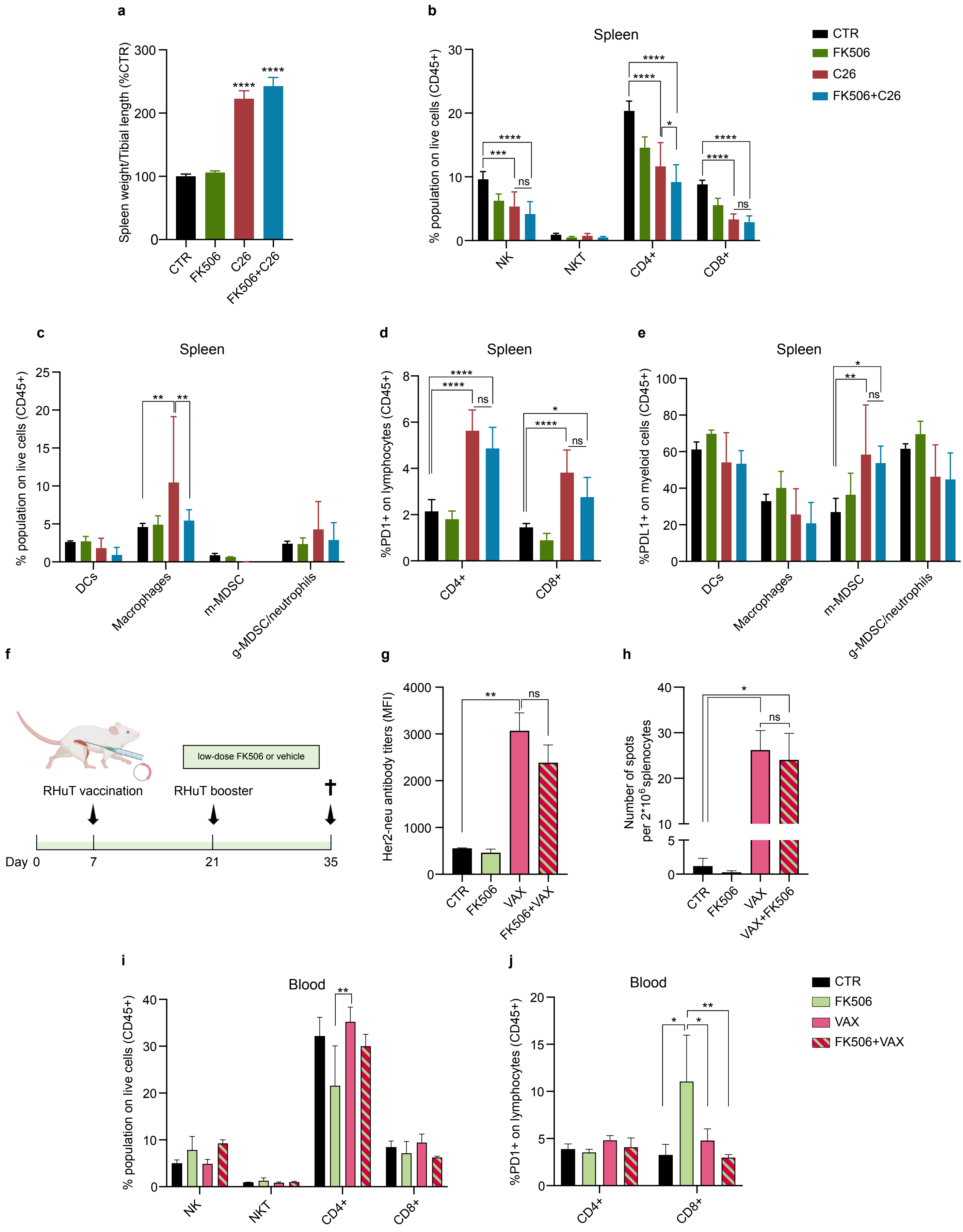

Figure S5: FK506 effect on the immune system *in vivo*. Related to Fig. 4.

a) Spleen weights normalized for tibial length in C26 tumor-bearing mice treated with FK506 and relative control groups. (n=11) **b-c)** Percentages of lymphoid (**b**) and myeloid (**c**) populations in the spleen of tumor and non-tumor bearing mice, treated or not with FK506. (n=4/6) **d-e)** Percentages of PD-1+ lymphocytes (**d**) and PD-L1+ myelocytes (**e**) in the spleens. (n=4/6) **f)** Experimental design of long-term low-dose FK506 in non-tumor bearing mice immunized with RHuT vaccine. BALB/c mice were pre-treated for 1 week with FK506 (0.02mg/Kg) before being vaccinated with RHuT plasmid or empty vector. Vaccination was repeated after 2 weeks, and mice were euthanized 35 days after the beginning of the experiment. All mice were daily treated with FK506 or vehicle through oral gavage. **g)** Titers of anti-Her2-neu antibodies (mean fluorescence intensity) in the sera of mice vaccinated (VAX) or not and treated with FK506 or vehicle. (n=3/5) **h)** ELISpot assay performed on splenocytes isolated from vaccinated and unvaccinated mice to measure IFN-γ-secreting cytotoxic T cells activated by the immunodominant peptide for ErbB2. (n=3/4) **i)** Amounts of lymphoid populations in the blood of vaccinated (VAX) or unvaccinated mice treated with chronic low-dose FK506 or vehicle. (n=3/5) **j)** Percentages of PD-1+ lymphocytes in the blood of the experimental groups of Fig. S5g. (n=3/5)

Data information: Statistical significance was tested with one-way ANOVA followed by Sidak's multiple comparison test in graph a, g, h; with two-way ANOVA in graphs b-e, i, j.

Supplementary Figure 6

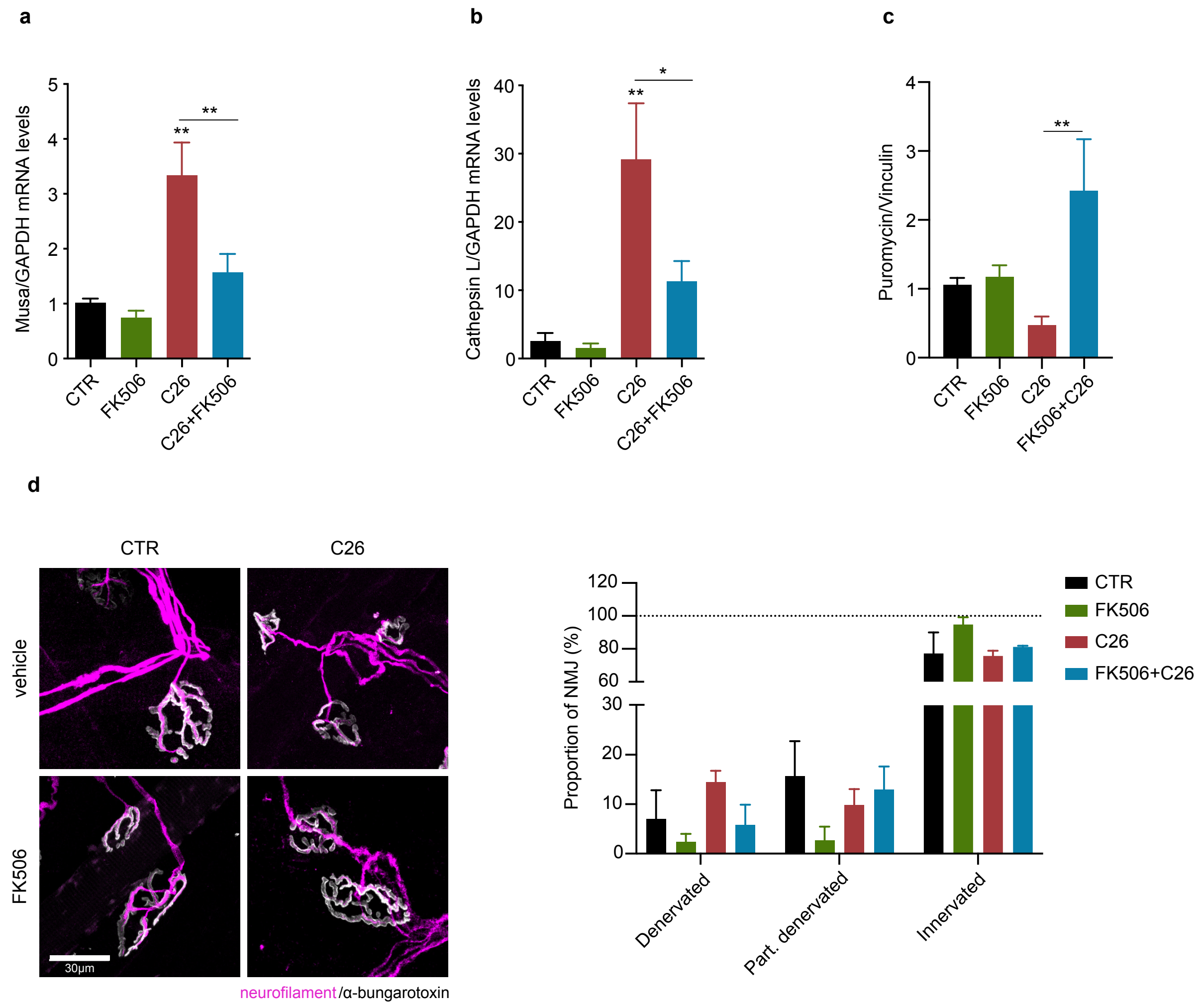

Figure S6: FK506 protects from cachexia, prevents the degeneration of neuromuscular junction morphology, and preserves muscle strength in C26 tumor-bearing mice. Related to Fig. 5 and Fig. 6.

a-b) Expression levels of the atrogenes Musa (**a**) and Cathepsin L (**b**) in the gastrocnemii of C26 and control mice treated with low-dose FK506 or vehicle. (n=4/6) **c)** Densitometric quantification of the *in vivo* SuNSET assay reported in Fig. 5h. (n=3/4). **d)** Representative immunofluorescence pictures and relative quantification of EDL muscles stained for α-bungarotoxin and anti-Neurofilament antibody to detect NMJs innervation. The quantification of denervated NMJs is expressed as percentage of the total number of NMJs quantified (60 to 90 NMJs per mouse, n=3 per group).

Data information: Statistical significance was tested with one-way ANOVA followed by Sidak's multiple comparison test in all graphs.

Video S1) FK506 protects from cachexia in C26 tumor-bearing mice. Related to Fig. 5.

C26-tumor bearing mice treated with vehicle (left) or FK506 (right) at day 11 post-C26 injection. Related to Fig. 5, 6 and Fig. S6.

Table S1

Gene	Species	Forward	Reverse
Atrogin1	Mouse	ATGCACACTGGTGCAGAGAG	TGTAAGCACACAGGCAGGTC
Murf1	Mouse	GTGTGAGGTGCCTACTTGCTC	GCTCAGTCTTCTGTCCTTGGA
Musa1	Mouse	TCGTGGAATGGTAATCTTGC	CCTCCCGTTTCTCTATCACG
Cathepsin L	Mouse	GTGGACTGTTCTCACGCTC	TCCGTCCTTCGCTTCATAGG
FKBP12	Mouse	ATGGGAGTGCAGGTGGAG	TCTTTCCATCTTCAAGCATCC
ERFE	Mouse	TGCTTGGATGCTGTTCGTCAA	CAGATGGGATAAAGGGGCCTG
GAPDH	Mouse	AGGTCGGTGTGAACGGATTTG	TGTAGACCATGTAGTTGAGGTCA
BMP6	Mouse	GGGATGGCAGGACTGGATCA	ATGGTTTGGGGACGTACTCG
BMP2	Mouse	CATCACGAAGAAGCCGTGGA	TGAGAAACTCGTCACTGGGG
BMP4	Mouse	TTCCTGGTAACCGAATGCTGA	CCTGAATCTCGGCGACTTTTT
Chrne	Mouse	GTGTCTGGATTGGCATTGACT	ACACCTGCAAAATCGTCCTTG
ERFE	Human	GGCCAAGAAGCTGAAGTTCGG	CTGCCGCACCGCACCTTTC
Beta Actin	Human	GGGAAATCGTGCGTGACA	GGACTCCATGCCCAGGA
ChIP-qPCR Erfe promoter	Mouse	GGGTGGATTGGTAGCCTTCA	TGCCTTGCAGAGGGAGGTAT
ChIP-qPCR Fkbp12 promoter	Mouse	ATTCGTGGAAGAGTGGGCTT	TGCCCTGTAAGGACGATAGC
ChIP-qPCR Negative Ctr	Mouse	TGGCCCCATAGGCTCATCTA	GGCCTGGCATGAGAGACTTT

Table S1: RT-qPCR SYBR primers and ChIP-qPCR primers. Related to STAR Methods.
